# Integrin beta3 regulates clonality and fate of smooth muscle-derived atherosclerotic plaque cells

**DOI:** 10.1038/s41467-018-04447-7

**Published:** 2018-05-25

**Authors:** Ashish Misra, Zhonghui Feng, Rachana R. Chandran, Inamul Kabir, Noemi Rotllan, Binod Aryal, Abdul Q. Sheikh, Ling Ding, Lingfeng Qin, Carlos Fernández-Hernando, George Tellides, Daniel M. Greif

**Affiliations:** 1 Yale Cardiovascular Research Center, Section of Cardiovascular Medicine, Department of Internal Medicine, Yale University School of Medicine, New Haven, CT 06511 USA; 2 Department of Genetics, Yale University School of Medicine, New Haven, CT 06511 USA; 3 Department of Comparative Medicine, Yale University School of Medicine, New Haven, CT 06511 USA; 4 Department of Surgery, Yale University School of Medicine, New Haven, CT 06511 USA; 5 Present Address: Pfizer, 610 Main Street, Cambridge, MA 02139 USA; 6 Present Address: Institute of Pharmacology and Toxicology, College of Pharmaceutical Science, Zhejiang University, Hangzhou, China

## Abstract

Smooth muscle cells (SMCs) play a key role in atherogenesis. However, mechanisms regulating expansion and fate of pre-existing SMCs in atherosclerotic plaques remain poorly defined. Here we show that multiple SMC progenitors mix to form the aorta during development. In contrast, during atherogenesis, a single SMC gives rise to the smooth muscle-derived cells that initially coat the cap of atherosclerotic plaques. Subsequently, highly proliferative cap cells invade the plaque core, comprising the majority of plaque cells. Reduction of integrin β3 (Itgb3) levels in SMCs induces toll-like receptor 4 expression and thereby enhances Cd36 levels and cholesterol-induced transdifferentiation to a macrophage-like phenotype. Global *Itgb3* deletion or transplantation of *Itgb3*^(−/−)^ bone marrow results in recruitment of multiple pre-existing SMCs into plaques. Conditioned medium from Itgb3-silenced macrophages enhances SMC proliferation and migration. Together, our results suggest SMC contribution to atherogenesis is regulated by integrin β3-mediated pathways in both SMCs and bone marrow-derived cells.

## Abstract (web summary)

Smooth muscle cells (SMCs) invade atherosclerotic lesions and expand, contributing to plaque progression. Here Misra et al. show that SMC-derived plaque cells come from a single SMC and integrin β3 in SMCs and macrophages regulate the fate, expansion and migration of SMCs during plaque formation.

## Introduction

Atherosclerosis is a primary cause of devastating diseases of diverse arterial beds, including the coronary arteries, cerebrovasculature, peripheral arteries, and the aorta. The atherosclerotic plaque or atheroma is the culprit lesion and consists of a lipid-laden core covered by a fibrous cap^1^. Rupture of the plaque results in thrombosis with dire consequences, such as myocardial infarction and stroke. Smooth muscle cells (SMCs) and macrophages are key players in atherogenesis, and a reduced ratio of SMCs to macrophages is associated with an unstable plaque that is prone to rupture.

Although the role of SMCs in atherogenesis of the aorta and its major branches has recently received increased attention^2–6^, many of the key processes and underlying mechanisms remain to be elucidated. An in depth understanding of the morphogenesis of SMC layers in the normal aortic media would aid in the investigation of SMC pathogenesis in vascular disease, but little is understood regarding normal aortic development. In advanced atherosclerotic plaques of high fat-fed *ApoE*^(−/−)^ mice, recent studies indicate pre-existing SMCs give rise to alpha-smooth muscle actin (SMA)^+^ cells and predominantly SMC marker^−^ cells, including cells expressing macrophage markers LGALS3 and CD68^2, 3^. Furthermore, deletion of the pluripotency factor Kruppel-like factor 4 (*Klf4*) in SMCs attenuates the expression of LGALS3^3^; however, underlying mechanisms are not well established. Interestingly, in mature human atheromas, 40% of CD68^+^ cells are also labeled by SMA^7^, and cholesterol loading of cultured SMCs induces downregulation of SMC markers and upregulation of macrophage markers^8, 9^. These studies attest to the importance of pre-existing SMCs in atherogenesis, but a temporospatial analysis of the dynamics of SMC fate and accumulation, which promises to yield key insights, is lacking. In addition, the clonal architecture of smooth muscle-derived cells in plaques is controversial^2, 10–15^ and has major ramifications on pathogenesis and potentially on therapeutic approaches: distinct strategies may be pursued to tailor therapies to target multiple progenitors (i.e., polyclonal plaque) or instead a single progenitor (i.e., monoclonal plaque). The roles of non-smooth muscle cells in modulating SMC fate and clonality have not been investigated, and no molecular or cellular regulators of SMC clonal patterning have been identified.

Integrins are heterodimeric transmembrane proteins, composed of α and β subunits, that link the extracellular matrix to the cytoskeleton, and global deletion of *Itgb3* (encoding integrin β3) exacerbates atherosclerosis in high fat-fed *ApoE*^(−/−)^ mice^16^. Although mechanisms underlying this exacerbation are not well established, *Itgb3*^(−/−)^ macrophages have been implicated^17^. In addition to hematopoietic cells (i.e., macrophages and platelets), β3 is highly expressed in SMCs, and we and other have demonstrated that β3 is critical for enhanced SMC proliferation and migration in vascular disease^18–20^. Treatment of arterial SMCs with an α5β1 antagonist attenuates fibronectin-induced SMA downregulation^21^, but the role of β3 in SMC differentiation or transdifferentiation is not known.

Herein, we use multi-color genetic labeling and single cell resolution clonal analysis to demonstrate that the normal aortic tunica media forms by mixing of multiple SMC progenitors. However, in *ApoE*^(−/−)^ mice, a single SMC that is present prior to the onset of high fat feeding gives rise to the majority of cells in an advanced atherosclerotic plaque. Furthermore, these studies identify cap cells in the early plaque as specialized cells that derive from a single pre-existing SMC and have a unique molecular signature, expressing markers of SMCs (SMA, smooth muscle myosin heavy chain (SMMHC)) and undifferentiated mesenchyme (platelet-derived growth factor receptor (PDGFR)-β), as well as integrin β3. In human and murine plaques, cap cells are highly proliferative, and early cap cells give rise to progeny that downregulate SMC markers and β3, and migrate into the core. Reduction of β3 stimulates SMC transdifferentiation by enhancing toll-like receptor 4 (Tlr4) and Cd36 expression, oxidized (ox)-LDL uptake, and cholesterol-induced downregulation of SMC differentiation markers and upregulation of macrophage markers. In addition, in the *ApoE*^(−/−)^ background, in contrast to *Itgb3* wild-type mice, global *Itgb3* deletion, or transplant of *Itgb3*^(−/−)^ bone marrow results in recruitment of multiple pre-existing SMCs into plaques. Finally, in comparison to *ApoE*^(−/−)^ macrophage-conditioned medium, medium conditioned by *ApoE*^(−/−)^, *Itgb3*^(−/−)^ macrophages induces SMC migration and proliferation. Thus, through both cell autonomous and non-cell autonomous effects, β3 regulates key factors—the number of SMCs recruited into the plaque and their fate—that dictate the course of atherosclerosis, and further investigation into regulation of these processes is needed.

## Results

### Multiple progenitors mix extensively to form the aortic wall

**Fig. 1:**
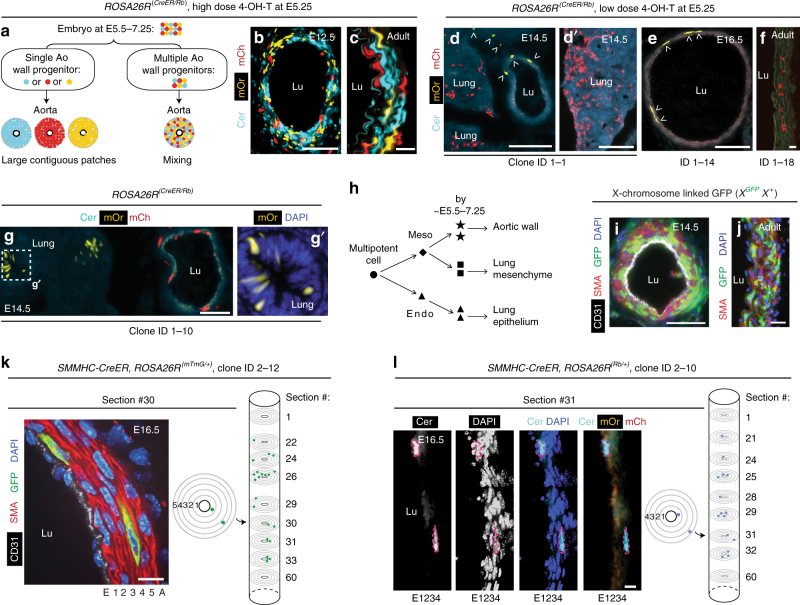
Aortic SMCs are polyclonal and highly migratory in development. **a** Cells of *ROSA26R*^(*CreER*/*Rb*)^ embryo induced with high dose 4-OH-T at E5.25 are expected to be of multiple colors at E5.5–7.25. If the aortic wall derives from a single cell (or instead from multiple polyclonal cells) present at ~E7.25 or thereafter, wall cells will be a single color (or multiple colors). **b**–**g** Sections of *ROSA26R*^(*CreER*/*Rb*)^ mice in transverse (**b**–**e**, **g**) or longitudinal axis (**f**) imaged with direct fluorescence of Rb channels. At E5.25, dams were induced with a single high 4-OH-T dose in **b**, **c** (150 µg) or, to label clonally, at a limiting dose in **d**, **d′** (100 µg; Supplementary Table 1, clone ID1-1), **e** (20 µg; ID1-14), **f** (20 µg; ID1-18), and **g** (50 µg; ID1-10). Arrowheads indicate mOrange^+^ cells (**d**, **e**), with marked cells in **d** suggesting ventral migration of aortic progenitors. By the time of recombination, progenitors of aorta and lung mesenchyme (**d**, **d′**) or epithelium (**g**, **g′**) are differentially specified (**h**) (Supplementary Table 1). **i**, **j** Transverse sections of *X*^*GFP*^*X*^+^ aorta stained for GFP at E14.5 and in the adult (5 weeks), representative of *n* = 4 embryos and 6 adults. **k**, **l** *SMMHC*-*CreER*^*T2*^ embryos also carrying *ROSA26R*^(*mTmG*/+)^ or *ROSA26R*^(*Rb*/+)^ induced with tamoxifen 0.5 mg at E9.5 (when there is one SMC layer; Supplementary Fig. 1b, c) and analyzed at E16.5. In **k** transverse aortic section (left panel, section #30) of a 21-cell GFP-marked clone (Supplementary Table 2, clone ID2-12; Supplementary Fig. 3) after GFP staining. The middle panel shows positions of two marked cells in this section, and columnar clone schematic (right panel) is a compilation of transverse sections, among 60 consecutive sections analyzed, that contain marked cells. The first and 60th sections are included for reference. A 15-cell clone (ID2-10) is shown in **l** with the section stained for DAPI and imaged for Rb colors. The clones in **k**, **l** expanded radially, circumferentially, and longitudinally. Lu, aortic lumen; E, endothelial layer; 1–5, smooth muscle layers; A, adventitial layer. Scale bars, 100 µm (**b**, **d**, **e**, **g**, **i**) and 10 µm (**c**, **f**, **j**–**l**)

Based on PCR analysis of X-chromosome inactivation patterns (as a crude clone marker) in micro-dissected adult human descending aorta, previous studies have suggested that the arterial wall is composed of large clonal SMC patches^11, 12^. Herein, we initially focused on the development of the murine descending aorta (Supplementary Fig. 1), and used single cell resolution clonal analyses and modern genetic tools to determine whether the progeny of individual early progenitor cells is cohesive or dispersed (Fig. 1). A single high dose (150 µg) of 4-hydroxy tamoxifen (4-OH-T) in dams pregnant with embryos carrying the multi-color ROSA26R-Rainbow (Rb) Cre reporter^22^ and the ubiquitous ROSA26R-CreER^23^ at embryonic day (E) 5.25 (the earliest time that reproducibly yields viable embryos) results in substantial intermixing of cells of different colors (i.e., polyclonal) in the thoracic descending aortic wall in *ROSA26R*^(*CreER*/*Rb*)^ mice at E12.5 or during adulthood (Fig. 1a–c). Furthermore, a threshold dose (20–100 µg) of 4-OH-T at E5.25 almost always results in an embryonic or adult descending aorta with either no labeled cells or all labeled cells of a single color (Fig. 1d–g and Supplementary Table 1; Methods). Thus, these marked cells, which have a diffuse pattern with unlabeled cells interspersed, derive from a single cell present at the time of recombination (estimated to be ~E5.5–7.25)^24, 25^. Indeed, the average patch size of contiguous marked cells in longitudinal sections of the adult distal ascending aorta is only 1.2 ± 0.1 cells (Supplementary Fig. 2). Therefore, at ~E5.5–7.25, there are multiple aortic wall progenitors, and the progeny of these progenitors mix extensively during embryogenesis, and this mixing persists. These findings are independently validated by our studies with female mice hemizygous for an X-chromosome-linked *GFP* transgene^26^ (Fig. 1i, j). In the early female mammalian embryo, one of two X-chromosomes in each somatic cell is randomly inactivated, and this process is complete by ~E7 in the mouse^26^. We observed a mixture of GFP^+^ and GFP^−^ cells in the embryonic and postnatal aorta suggesting that the descending aortic wall derives from multiple polyclonal cells present at E7 or thereafter. Finally, clonal analysis of initial inner layer SMC marker^+^ cells in the developing aorta indicates that their progeny migrate radially, longitudinally, and circumferentially within the media (Fig. 1k, l, Supplementary Fig. 3 and Supplementary Table 2; Methods).

Descending aortic SMCs derive from the presomitic mesoderm^27, 28^, and *ROSA26R*^(*CreER*/*Rb*)^ embryos induced by threshold doses of 4-OH-T at E5.25 often contained descending aortic SMCs marked with one color, as well as cells in an adjacent mesoderm-derived tissue (i.e., lung mesenchyme; Fig. 1d, d′ and Supplementary Table 1) or in an endoderm-derived tissue (i.e., lung epithelium; Fig. 1g, g′) marked with a single distinct color. Thus, at ~E5.5–7.25, the mesoderm and endoderm are differentially specified as are progenitors of the aortic wall and lung mesenchyme (derived from lateral plate mesoderm; Fig. 1h). Similar to the aorta, substantial migration and mixing of the progeny of early lung progenitors are indicated by the diffuse pattern of marked cells in the mesenchyme and epithelium.

### Most atherosclerotic plaque cells derive from one progenitor

**Fig. 2:**
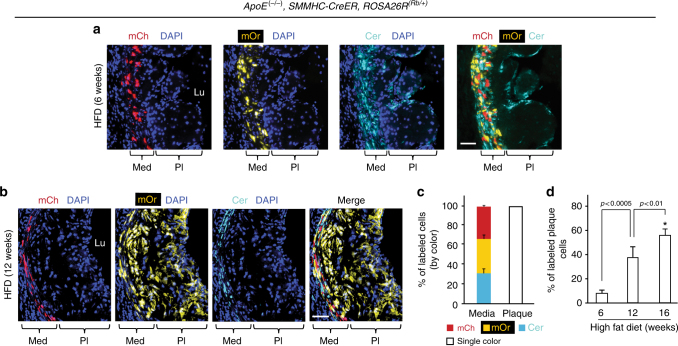
A single SMC gives rise to most of the cells in an atherosclerotic plaque. **a**, **b** *ApoE*^(−/−)^, *SMMHC*-*CreER*^*T2*^, *ROSA26R*^(*Rb*/+)^ mice were induced with tamoxifen (1 mg/day for 5 days), rested, fed a high fat diet (HFD) for 6 or 12 weeks as indicated, and transverse aortic root sections were stained with DAPI and directly imaged for the Rb colors (mCherry (mCh), mOrange (mOr), and Cerulean (Cer)). Marked cells in each atherosclerotic plaque are a single color (Cer in **a** and mOr in **b**) indicating that a single SMC gives rise to all smooth muscle-derived plaque cells. Scale bars, 50 μm. **c** Quantification of marked cells by Rb color in aortic root atherosclerotic plaques and adjacent media mice fed a high fat diet for 6, 12, and 16 weeks from sections stained and imaged as in **a** and **b**. *n* = 9 mice (all male), 11 atherosclerotic plaques, 6 sections per plaque were quantified and the average number of labeled plaque cells scored per plaque by duration of high fat diet: 6 weeks (80 plaque cells), 12 weeks (458), and 16 weeks (936). **d** Quantification of the average percent of atherosclerotic plaque cells that are SMC-derived classified by weeks of high fat feeding. *n* = 9 mice, 11 plaques. *vs. 6 weeks, *p* < 0.0001. One-way ANOVA with Tukey’s multiple comparisons test was used. Error bars represent standard deviations

We next evaluated whether the extensive SMC progenitor migration and mixing, which occurs during aortic morphogenesis also characterizes the accumulation of excess SMC-derived cells during atherosclerosis. *ApoE*^(−/−)^, *ROSA26R*^(*Rb*/+)^ mice also carrying *SMMHC*-*CreER*^*T2*^ or *SMA*-*CreER*^*T2*^ were induced with tamoxifen (1 mg/day for 5 days), rested for 5 days, and fed a high fat diet (HFD) for 6, 12, or 16 weeks. Eleven aortic root atherosclerotic plaques were analyzed, and in ten plaques, all labeled cells were a single Rb color (Fig. 2a–c, Supplementary Fig. 4a and Supplementary Table 3), and in one plaque (at 16 weeks), 608 of the 613 labeled cells were Cerulean^+^ and the remaining 5 cells were mCherry^+^ (Supplementary Table 3). In terms of smooth muscle-derived cells, brachiocephalic plaques are similarly monoclonal (Supplementary Fig. 4b). With increasing HFD duration, labeled cells constituted a higher percentage of total aortic plaque cells (remarkably, 57 ± 7% by 16 weeks; Fig. 2d and Supplementary Table 3). Thus, a single pre-existing SMC is the source of the majority of cells of an advanced atherosclerotic plaque, and the smooth muscle progenitors undergo robust clonal expansion during plaque formation.

In addition to SMCs, macrophages play key roles in atherogenesis, but the clonal architecture of macrophages in plaques has not been studied. Mice carrying the inducible *Csf1r*-*Mer*-*iCre*-*Mer*^29^ were used to label monocytes or macrophages (Supplementary Fig. 5a). *ApoE*^(−/−)^, *Csf1r*-*Mer*-*iCre*-*Mer*, *ROSA26R*^(*Rb*/+)^ mice were induced with tamoxifen (1 mg/day for 20 days), rested, and then fed a HFD for 6–9 weeks. The resulting plaques have labeled cells of multiple colors (Supplementary Fig. 5b) suggesting that in contrast to SMCs, multiple CSF1R^+^ cells give rise to cells in atherosclerotic lesions.

### Timeline of the SMC lineage during atherogenesis

**Fig. 3:**
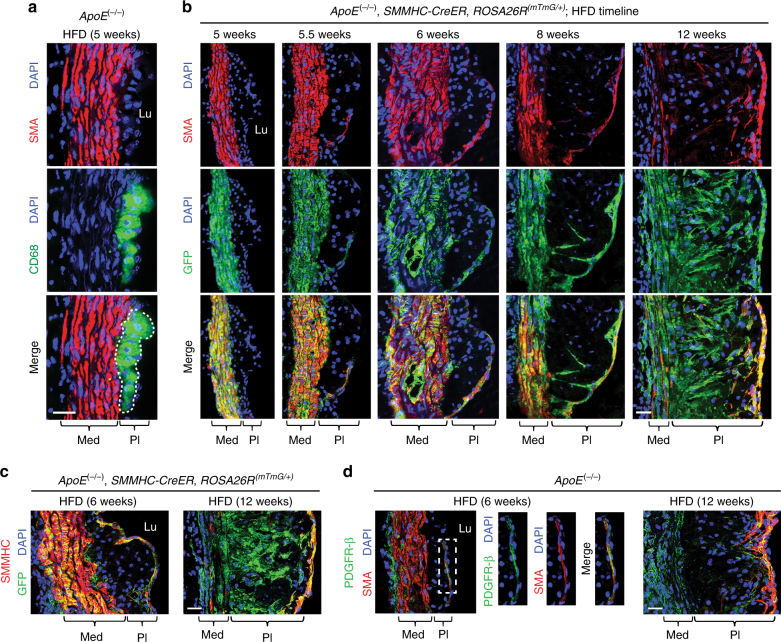
Smooth muscle-derived cells initially coat the cap of the plaque and then invade the core and dedifferentiate. Transverse aortic root sections of mice fed a HFD for 5–12 weeks as indicated were immunostained. **a**, **d** Sections from *ApoE*^(−/−)^ mice were stained for SMA, nuclei (DAPI), and either CD68 (**a**) or PDGFR-β (**d**). **b**, **c** Prior to high fat feeding, *ApoE*^(−/−)^, *SMMHC*-*CreER*^*T2*^, *ROSA26R*^(*mTmG*/+)^ were induced with tamoxifen, and sections were stained for GFP (fate marker), nuclei (DAPI), and either SMA (**b**) or SMMHC (**c**). Results are representative of *n* = 5 mice. Lu, aortic lumen; Med, tunica media; Pl, plaque. Scale bars, 25 μm

We next evaluated the temporospatial pattern of the contribution of SMC-derived cells to the atherosclerotic plaque in mice maintained on a HFD for increasing durations. After 5 weeks of high fat feeding, the plaques of *ApoE*^(−/−)^ mice contain many cells expressing CD68 (Fig. 3a). Based on fate mapping with *ApoE*^(−/−)^, *SMMHC*-*CreER*^*T2*^, *ROSA26R*^(*mTmG*/+)^ mice induced with tamoxifen and then maintained on a HFD, these early CD68^+^ cells do not derive from pre-existing SMCs (i.e., GFP^−^; Fig. 3b). SMC-derived cells (i.e., GFP^+^) expressing SMA initially enter the plaque at the shoulder of the cap at 5.5 weeks (Fig. 3b). By 6 weeks, GFP^+^SMA^+^SMMHC^+^ cells cover most of the cap, and SMA^+^ cap cells express the undifferentiated mesenchyme marker PDGFR-β (Fig. 3b–d and Supplementary Fig. 6). At 8 weeks, SMC-derived cells are present in the internal region (or core) of the plaque and many have downregulated SMC markers. This process continues and by 12 weeks of high fat feeding, a substantial portion of core cells are GFP^+^ and have low or no SMC marker expression (Fig. 3b). At this time point, GFP^+^ cells that cover the cap remain SMA^+^SMMHC^+^, and SMA^+^ cap cells continue to express PDGFR-β (Fig. 3b–d and Supplementary Fig. 6).

### Cap cells in human and murine plaques are hyperproliferative

**Fig. 4:**
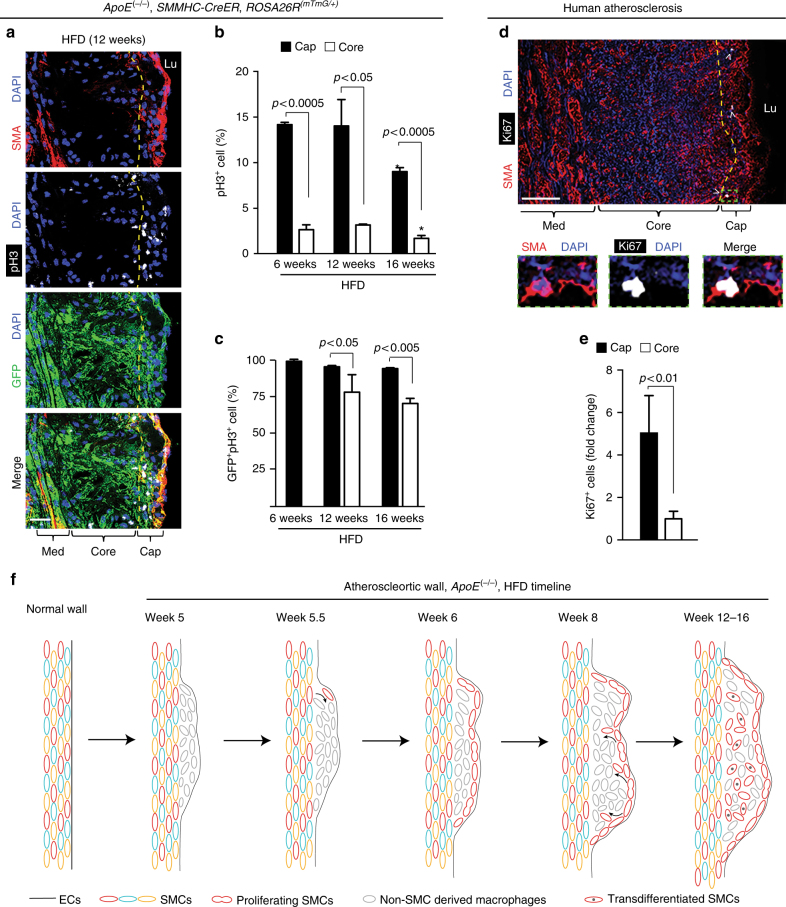
Cap cells and smooth muscle-derived cells in the plaque are highly proliferative. **a**–**c** *ApoE*^(−/−)^, *SMMHC*-*CreER*^*T2*^, *ROSA26R*^(*mTmG*/+)^ were induced with tamoxifen and fed a HFD for 6, 12, or 16 weeks. In **a** transverse aortic root sections were stained for phosphohistoneH3 (pH3; mitotic marker), GFP (fate marker), SMA, and nuclei (DAPI). In **b** percentage of total cap or core cells that are pH3^+^ is shown. *vs. 6, 12 weeks, *p* < 0.05. In **c** percentage of pH3^+^ cells in the cap or core that are GFP^+^ is displayed. Percentages in **b**, **c** are quantified from images as shown in **a**; *n* = 3 mice for each time point and 10 sections per mouse. One-way ANOVA with Tukey’s multiple comparisons test and Student’s *t*-test were used. Per convention, the cap is defined as plaque region within 30 μm of lumen boundary^3^ for mice maintained on HFD for 12 or 16 weeks and within 15 μm for 6 weeks of HFD (see Methods). Cap cells and smooth muscle-derived plaque cells are highly proliferative. **d** Section of atherosclerotic left main coronary artery from a 64-yr-old man stained for proliferation marker Ki67 (arrowheads), SMA, and nuclei (DAPI). Region in green box is shown below as close-ups. **e** Average of total Ki67^+^ cells in core or cap compartment per human subject relative to area of the compartment and normalized to this quotient for the core. Quantification was conducted on images as shown in **d**. A total of 84 Ki67^+^ cells from atherosclerotic artery samples of six humans were scored for location. Student’s *t*-test was used. **f** Schematic depicts the accumulation of SMC-derived cells in atherosclerotic plaque. At 5.5 weeks of HFD in *ApoE*^(−/−)^ mice, a single SMC enters the cap at the shoulder. The lineage of this progenitor SMC is highly proliferative and rapidly covers the cap by 6 weeks. By 8 weeks, these SMC-derived cells begin to migrate into the core and by 12 weeks, most SMC-derived cells in the core have downregulated SMC markers. Error bars represent standard deviations. Lu, lumen; Med, tunica media; Pl, plaque. Scale bars, 25 μm (**a**), 100 μm (**d**)

Given the marked clonal expansion of a single smooth muscle-derived cell in atherogenesis, we evaluated cellular proliferation in the plaque. Our studies with human samples and high fat fed *ApoE*^(−/−)^ mice indicate that cap cells are ~5 times more proliferative than cells in the core (Fig. 4a, b, d, e). In addition, the large majority of proliferating cells are SMC-derived, both in the cap (94–99%) and in the core (70–78%) (Fig. 4c). Proliferation of GFP^+^ cap cells is independent of SMA expression as there is no difference in the percentage of pH3^+^ cells in SMA^+^GFP^+^ or SMA^−^GFP^+^ cells (Supplementary Fig. 7). Taken together, these data suggest that a single pre-existing SMC gives rise to highly proliferative SMC marker^+^ cells that initially coat the cap. In turn, these cap SMCs or their progeny migrate into the core and downregulate SMC markers (Fig. 4f, schematic).

### Reduced integrin β3 levels induces SMC transdifferentiation

**Fig. 5:**
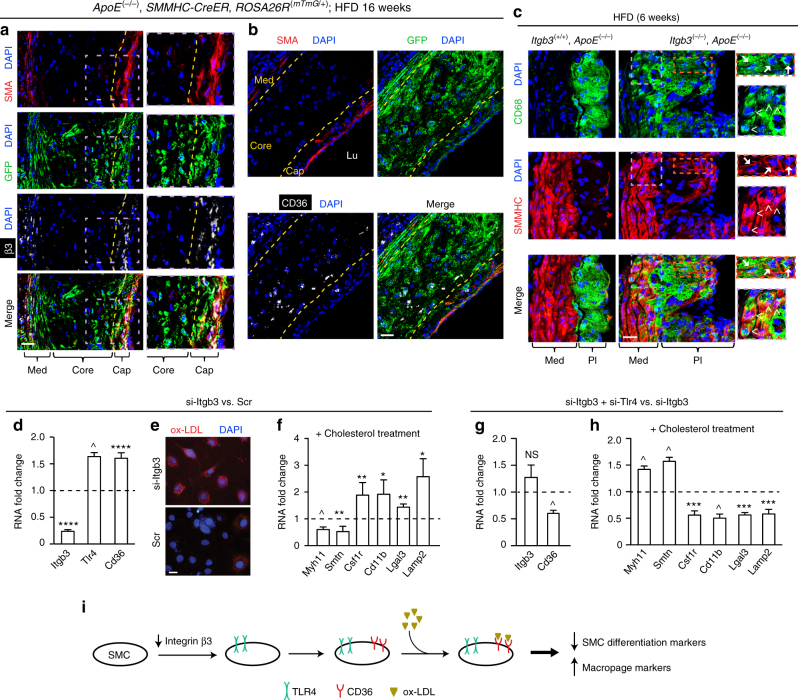
Integrin β3 modulates SMC transdifferentiation. **a**–**c** Mice were fed a HFD for 6 or 16 weeks as indicated, and then transverse aortic root sections were stained. In **a**, **b** sections from *ApoE*^(−/−)^, *SMMHC*-*CreER*^*T2*^, *ROSA26R*^(*mTmG*/+)^ mice were stained for SMA, GFP (fate marker), nuclei (DAPI), and either integrin β3 (**a**) or CD36 (**b**). Dashed yellow lines separate cap from core (**a**, **b**) and core from media (**b**). *n* = 5. In **c** sections from *ApoE*^(−/−)^ mice that were also wild type or null for *Itgb3* were stained for SMMHC, CD68, and nuclei (DAPI). *n* = 3. Boxed regions (**a**, **c**) are shown as close-ups on right; in **c** CD68^+^SMMHC^+^ cells in the media (arrowheads) and plaque (arrows) of the *Itgb3* null atherosclerotic aorta are indicated. Med, tunica media; Lu, lumen; Pl, plaque. Scale bars, 25 μm. **d**–**h** Aortic SMCs were isolated from *ApoE*^(−/−)^ mice and then subjected to siRNA-mediated knockdown with si-Itgb3 vs. scrambled (Scr; **d**–**f**) or with si-Itgb3, si-Tlr4 vs. si-Itgb3 (**g**, **h**). Levels of indicated transcripts from qRT-PCR are relative to Gapdh and normalized to either Scr in **d**, **f** or to si-Itgb3 in **g**, **h**. For **d**, **g**, *n* = 4–5 in duplicate. In **e** silenced SMCs were cultured with DiI-conjugated ox-LDL for 10 h and stained with DAPI; *n* = 5. In **f**, **h** silenced SMCs were exposed to soluble cholesterol:methyl-β-cyclodextrin complexes for 3 days, and then mRNA levels were assessed; *n* = 4–7 in duplicate. *^,^ **^,^ ***^,^ ^^,^ **** vs. control (Scr in **d**, **f** and si-Itgb3 in **g**, **h**), *p* < 0.05, *p* < 0.01, *p* < 0.005, *p* < 0.001, and *p* < 0.0005, respectively. NS, not significant. Student’s *t*-test was used, and error bars represent standard deviations. **i** Schematic of the effect of β3 reduction via TLR4 and CD36 in SMCs on transdifferentiation

We next sought molecular regulators that modulate SMC fate and recruitment to the atherosclerotic plaque. Integrins are heterodimeric receptors that link the extracellular matrix to the cytoskeleton, and deletion of the gene encoding integrin β3 (*Itgb3*) exacerbates atherosclerosis burden in atheroprone mice^16^. In high fat-fed *ApoE*^(−/−)^ mice, β3 is highly expressed in SMA^+^ cells of the tunica media and cap (that are derived from pre-existing SMMHC^+^ cells) but not in SMA^−^ core cells (Fig. 5a and Supplementary Fig. 8), which mimics the pattern of β3 expression in human atherosclerosis^30^. In contrast, in GFP^+^ (SMC-derived) cells of the advanced atherosclerotic plaque of *ApoE*^(−/−)^, *SMMHC*-*CreER*^*T2*^, *ROSA26R*^(*mTmG*/+)^ mice, the scavenger receptor CD36 is primarily expressed in the region of the core that is adjacent to the cap and not in GFP^+^ cells of the cap itself (Fig. 5b). In the background of high fat fed *ApoE*^(−/−)^ mice, *Itgb3* deletion results in expression of the macrophage marker CD68 in many SMMHC^+^ cells in the plaque and adjacent media of the atherosclerotic aorta (Fig. 5c and Supplementary Fig. 9).

Complementing these in vivo studies, reduction of Itgb3 in SMCs isolated from atheroprone mice increases Cd36 levels (Fig. 5d)^16^ but does not alter expression of other major cholesterol receptors/transporters Ldlr, Lrp1, Abca1 (Supplementary Fig. 10a). As a result, we observed an enhanced SMC uptake of ox-LDL (Fig. 5e). Itgb3 silencing does not alter Myh11 levels in isolated *ApoE*^(−/−)^ SMCs under basal conditions (Supplementary Fig. 10b); however, with cholesterol loading, in comparison to pre-treatment with scrambled siRNA, Itgb3 siRNA pre-treatment results in reduced levels of SMC differentiation markers Myh11 and Smtn, and increased levels of macrophage markers Csf1r, Cd11b, Lgals3, and Lamp2 (Fig. 5f ).

We next investigated further molecular mediators that may contribute to the effects of Itgb3 deficiency on the conversion of SMCs to macrophage-like cells. The pattern recognition receptor TLR4 and the pluripotency factor KLF4 have each been implicated in phenotypic changes of SMCs^3, 31^. siRNA-mediated knockdown of Itgb3 in aortic SMCs isolated from *ApoE*^(−/−)^ mice enhances Tlr4 levels (Fig. 5d) without altering levels of Klf4 (Supplementary Fig. 10b). Furthermore, on the background of Itgb3 silencing in *ApoE*^(−/−)^ SMCs, Tlr4 knockdown attenuates Cd36 levels (Fig. 5g) and with cholesterol loading, induces expression of SMC markers while attenuating macrophage marker levels (Fig. 5h). Thus, reduction of Itgb3 in SMCs increases Tlr4 levels, which facilitates Cd36 expression and cholesterol-induced transdifferentiation to a macrophage-like phenotype (Fig. 5i, schematic).

### β3 in bone marrow-derived cells regulates SMC clonality

**Fig. 6:**
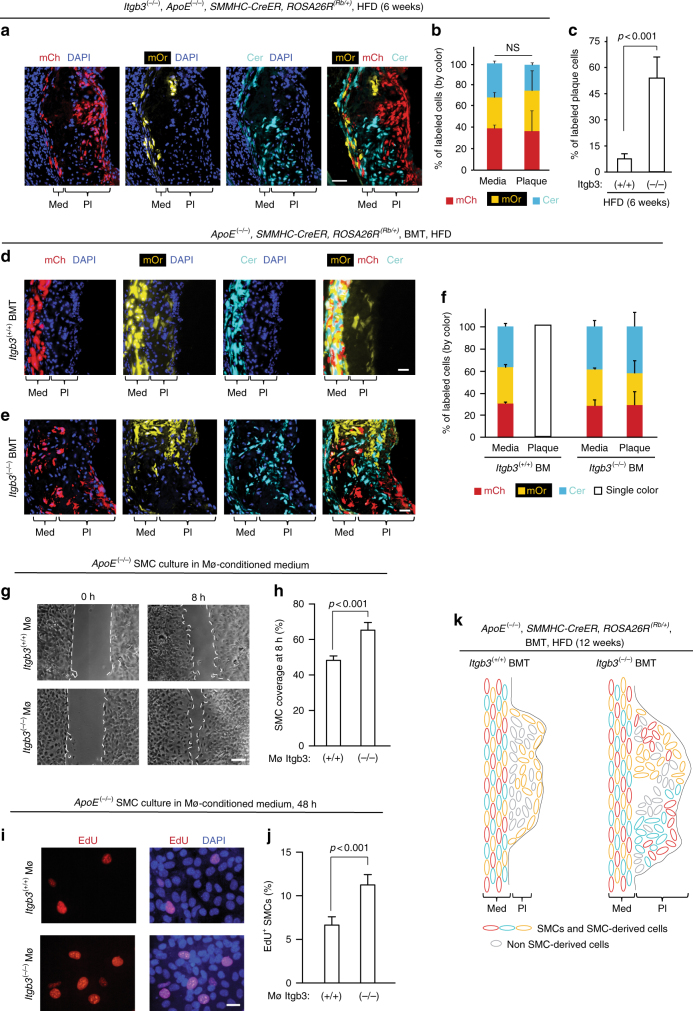
Integrin β3 in bone marrow-derived cells regulates SMC clonality. **a**, **b** *Itgb3*^(−/−)^, *ApoE*^(−/−)^, *SMMHC*-*CreER*^*T2*^, *ROSA26R*^(*Rb*/+)^ mice were fed a HFD for 6 weeks. In **a** aortic root sections were stained with DAPI and directly imaged for Rb colors. Note, multiple patches of smooth muscle-derived plaque cells of different colors with limited mixing contrasts with monoclonal plaques in *Itgb3*^(+/+)^ mice (Fig. 2a, b). In **b** percentage of labeled cells of each color in media and plaque is shown; *n* = 3 mice, 6 plaques. Chi-square test was used; NS, not significant. **c** Percent of plaque cells at 6 weeks of HFD labeled by any color were compared in *ApoE*^(−/−)^, *SMMHC*-*CreER*^*T2*^, *ROSA26R*^(*Rb*/+)^ mice that were also *Itgb3* wild type or null. *Itgb3*^(+/+)^ data is also shown in Fig. 2d. **d**–**f** *ApoE*^(−/−)^, *SMMHC*-*CreER*^*T2*^, *ROSA26R*^(*Rb*/+)^ mice were induced with tamoxifen, lethally irradiated, and transplanted with *Itgb3*^(+/+)^, *ApoE*^(−/−)^ (**d**) or *Itgb3*^(−/−)^, *ApoE*^(−/−)^ (**e**) bone marrow prior to HFD. In **d**, **e** after 12 weeks of HFD, aortic root sections were stained with DAPI and imaged for Rb colors. In **f** mice were fed a HFD for 6–12 weeks, and percentage of labeled cells of each Rb color in media and plaque were quantified; *n* = 5 recipient mice, 6 plaques for each donor genotype. **g**–**j** *ApoE*^(−/−)^ SMC were cultured in medium conditioned by bone marrow-derived macrophages from *Itgb3*^(+/+)^, *ApoE*^(−/−)^ or *Itgb3*^(−/−)^, *ApoE*^(−/−)^ mice for 0 and 8 h or 48 h. In **g**, **h** bright-field images and percent of SMC coverage at 8 h of uncovered area at 0 h are shown, respectively; *n* = 4. In **i**, **j** EdU was added to culture medium for the last 8 h of the 48 h incubation, and then SMCs were stained for EdU and DAPI and percent of cells expressing EdU was quantified; *n* = 4. Student’s *t*-test was used. **k** Schematics of plaques in *ApoE*^(−/−)^, *SMMHC*-*CreER*^*T2*^, *ROSA26R*^(*Rb*/+)^ mice that are *Itgb3* wild type or null were induced with tamoxifen, transplanted with *ApoE*^(−/−)^ bone marrow fed a HFD for 12 weeks. Error bars represent standard deviations. Med, tunica media; Pl, plaque. Scale bars, 25 μm (**a**, **d**, **e**, **i**), 100 μm (**g**)

In addition to SMC fate, we found that in contrast to the monoclonal plaques in *Itgb3* wild-type mice (see Fig. 2a–c and Supplementary Fig. 4), *Itgb3*^(−/−)^, *ApoE*^(−/−)^, *SMMHC*-*CreER*^*T2*^, *ROSA26R*^(*Rb*/+)^ mice fed a HFD have polyclonal SMC-derived cells in the atherosclerotic plaque as evidenced by multi-color labeling of plaque cells (Fig. 6a, b, Supplementary Fig. 11 and Supplementary Table 4). The multiple patches of cells of distinct colors with minimal mixing suggest that multiple SMC progenitors enter the plaque and then clonally expand within the plaque with limited migration. At 6 weeks of high fat feeding, labeled cells constitute a ~7-fold higher percentage of total plaque cells in *Itgb3* null mice, compared to that of wild-type *Itgb3* mice (56 ± 11% vs. 8 ± 3%; Fig. 6c).

Based on these results with global *Itgb3* null mice, as well as a previous study demonstrating that transplant of *Itgb3*^(−/−)^ bone marrow into *Itgb3*^(+/+)^ atheroprone mice worsens atherosclerosis in high fat fed mice^17^, we postulated that *Itgb3*^(−/−)^ bone marrow-derived cells induce multiple SMCs to migrate into the plaque. To test this hypothesis, bone marrow was harvested from experimental *Itgb3*^(−/−)^, *ApoE*^(−/−)^ or control *Itgb3*^(+/+)^, *ApoE*^(−/−)^ donor mice, and transplanted into *Itgb3*^(+/+)^, *ApoE*^(−/−)^, *SMMHC*-*CreER*^*T2*^, *ROSA26R*^(*Rb*/+)^ recipient mice. There are similar numbers of total leukocytes, neutrophils, lymphocytes, macrophages, red blood cells, and platelets in the peripheral blood of experimental and control donors (Supplementary Fig. 12). Recipient mice were induced with tamoxifen, rested, irradiated, and then transplanted with experimental or control bone marrow. Four weeks after transplant, recipients were initiated on a HFD for 6–12 weeks. Similar to untransplanted *Itgb3* wild-type mice (see Fig. 2a–c and Supplementary Fig. 4), plaques of recipient mice transplanted with control bone marrow contain a single Rb color, indicating that a single pre-existing SMC is recruited into the plaque (Fig. 6d, f). In contrast, recipient mice transplanted with experimental bone marrow have multi-color plaques, indicating polyclonality of SMC-derived cells (Fig. 6e, f ).

These findings suggest that bone marrow-derived cells and most likely macrophages regulate the recruitment of SMC progenitors into the developing atherosclerotic plaque. To evaluate this hypothesis further, monocytes from the femurs of experimental *Itgb3*^(−/−)^, *ApoE*^(−/−)^ or control *Itgb3*^(+/+)^, *ApoE*^(−/−)^ mice were harvested and differentiated into macrophages in culture. Isolated *ApoE*^(−/−)^ aortic SMCs were then cultured in the presence of medium conditioned by these experimental or control macrophages and subjected to migration and proliferation assays. During the first 8 h of culture, experimental macrophage conditioned medium induces *ApoE*^(−/−)^ SMC migration (Fig. 6g, h) without altering proliferation (Supplementary Fig. 13). Subsequently, *ApoE*^(−/−)^ SMCs cultured with experimental conditioned medium for two days are more proliferative than those cultured in control medium (Fig. 6i, j). In sum, β3 is required in bone marrow-derived cells (and probably in macrophages specifically) for limiting the number of source SMCs recruited into an atherosclerotic plaque to a single cell (Fig. 6k, schematic) as *Itgb3*^(−/−)^ macrophages enhance SMC migration and proliferation.

## Discussion

Herein, we use clonal analysis to compare and contrast the biology of SMC progenitors in the morphogenesis of the normal aortic wall and the pathogenesis of atherosclerosis. Based on our multiple independent single cell resolution clonal analyses in the mouse, we conclude that normal embryonic and adult aortic wall cells derive from multiple progenitors that are present at the time of gastrulation and whose progeny undergo robust mixing and migration (Fig. 1). Classical X-chromosome-linked inactivation studies of micro-dissected aortic segments from adult women heterozygous for genes on the X-chromosome, initially used zymography and subsequently PCR analysis as a crude clone marker^10–12^. PCR results demonstrated that ~25% of samples from the normal aortic media were skewed to the same allele and were interpreted as suggesting that the aorta forms by expanding coherent smooth muscle clones with minimal mixing of adjacent clones^11, 12^. The difference between the conclusions in regard to normal aortic media morphogenesis of these human studies and our high-resolution investigations in mice likely reflect inherent limitations of the human studies and/or differences between species.

In addition to vessel development, we studied the clonal architecture of cells derived from monocytes/macrophages or SMCs in atherosclerotic plaques. Although macrophages largely accumulate in plaques through local proliferation^32^, our results suggest that multiple macrophages are recruited into the plaque (Supplementary Fig. 5). In contrast, our studies utilizing *ApoE*^(−/−)^, *SMMHC*-*CreER*^*T2*^, *ROSA26R*^(*Rb*/+)^ mice fed a HFD for 6, 12, or 16 weeks demonstrate that in 91% (10 out of 11) of the atherosclerotic plaques analyzed, a single SMC gives rise to all smooth muscle-derived cells which comprise the majority of the total cells in an advanced plaque (Fig. 2). The one oligoclonal plaque observed was a 16-week plaque with two SMC progenitors, one of which dominated giving rise to more than 99% of SMC-derived cells (Supplementary Table 3).

These results must be considered in light of other investigations as the clonality of SMC-derived cells in atherosclerotic plaques is controversial^2, 10–15^. For instance, the authors of a study of tritiated thymidine labeling patterns in high cholesterol fed swine interpreted their results as indicating polyclonal plaques^13^. In contrast, a study analyzing X-chromosome inactivation in adult women initially indicated that most aortic atheromas are composed of large monoclonal SMC patches^10^; however, because the normal human aortic wall was subsequently suggested to be composed of large patches of SMCs with the same X-chromosome inactivation pattern (see above), such approaches cannot distinguish whether a single SMC or multiple SMCs located in proximity in the normal wall give rise to plaque cells^11, 12^. In addition, low-resolution X-gal images of fate-mapped cells expressing the SMC marker SM22α in 1-year-old normal chow-fed *ApoE*^(−/−)^ mice suggest that clonal expansion of a single pre-existing SM22α^+^ cell may contribute to regions of plaques^2^.

Most recently, two studies of *SMMHC*-*CreER*^*T2*^ mice also carrying the multi-color ROSA26R(Confetti) Cre reporter^33^ in atheroprone models have analyzed the clonal architecture of SMC-derived plaque cells^14, 15^. In the first study, these mice were on an *ApoE*^(−/−)^ background, induced with tamoxifen (10 mg) over two weeks and then fed a HFD for 16–19 weeks^14^. One-half of the plaques contained SMC-derived cells of a single color (monoclonal) and the other half contained SMC-derived cells of two or, in 8% of cases, more colors (oligoclonal or polyclonal)^14^. In the second study, the mice were induced with tamoxifen (1 mg/day for 10 days) and then infected with rAAV8-mD377Y-mPCSK9 virus particles and maintained on a HFD for 12, 24, or 36 weeks^15^. The authors concluded that all SMC-derived plaque cells result “from a very limited number of medial SMCs undergoing coherent oligoclonal proliferation”^15^. The different results between these recent studies and our study are likely due to the atherosclerosis model, the HFD duration in *ApoE*^(−/−)^ mice as all plaques that we analyzed at 6 or 12 weeks (*n* = 7) were monoclonal and/or a difference between the Confetti and Rb reporters. Indeed, the Confetti reporter alters PCSK9/HFD-induced atherosclerosis burden^15^. This Cre reporter includes multiple loxP sites that are oriented in opposite directions, and thus, Cre will invert the intervening sequence resulting in the expression of a different fluorophore^33, 34^. Hence, if proliferation occurs in a labeled parental SMC progenitor during the tamoxifen phase, the parental and daughter cells may have distinct colors. In contrast, the Rb reporter utilized in our studies contains pairs of incompatible loxP sites in the same orientation such that only a single mutually exclusive recombination event can occur, resulting in the indelible expression of a single color^22, 35, 36^.

Thus, taking together the results of our study and those of other studies also using modern genetic marking approaches, multiple SMC progenitors mix and migrate widely in the morphogenesis of the normal aortic wall, whereas a single (or at most, rare) SMC marker^+^ cell(s) in the adult *ApoE*^(−/−)^ aorta are recruited into the early atherosclerotic plaque and clonally expands. However, the SMC marker^+^ progenitor cell or pool of cells that is recruited into the early atherosclerotic plaque has not been identified in the aortic media. We previously identified in the normal lung, a pool of specialized SMC progenitors located at the muscular–unmuscular arteriole border, and with hypoxia exposure, one of these progenitors migrates distally and clonally expands giving rise to pathological distal arteriole SMCs^36^. Further studies are needed to evaluate whether there is a similar specialized pool of rare SMC marker^+^ progenitors in the normal aortic wall that are capable of being recruited into and clonally expanding in early adjacent plaques upon high fat feeding. An alternative hypothesis is that a progenitor SMC is stochastically selected among a level playing field of SMMHC^+^ cells to enter the plaque and proliferate, and this cell and/or its progeny exert an inhibitory influence on other medial SMCs preventing them from entering the plaque. If a pool of SMC progenitors that expand in atherosclerosis is identified, therapies targeting such cells have far-reaching implications.

Similar to our prior studies of hypoxia-induced pulmonary arteriole remodeling^36, 37^, herein, a time course of smooth muscle-derived cells during HFD-induced atherosclerosis reveals critical insights into pathogenesis (Fig. 3). The cells of the early atherosclerotic plaque are CD68^+^ and do not derive from SMCs but instead likely come from monocyte recruitment and/or perhaps local macrophage proliferation in the plaque^32^. Subsequently, the lineage of a single SMC covers the cap. These cap cells have a unique molecular signature expressing markers of SMCs (SMA, SMMHC) and undifferentiated mesenchyme (PDGFR-β) (Fig. 3 and Supplementary Fig. 6), and our studies of both human and murine plaques indicate that the cap cells are 5-fold more proliferative than core cells (Fig. 4). After giving rise to SMC marker^+^ cap cells, smooth muscle-derived cells populate the core of the plaque, and core cells have low expression of SMC markers. These results suggest that the smooth muscle-derived SMC marker^+^ cap cells are the source of many of the SMC marker^−^ cells in the internal region of the plaque. In previous fate mapping studies of 18-week high fat fed *ApoE*^(−/−)^ mice, Shankman et al. reported that 82% of SMC-derived cells assume SMA^−^ fates, including LGALS3^+^ macrophage-like cells, in atherosclerotic plaques^3^. Furthermore, deletion of *Klf4* in SMCs attenuated their transdifferentiation to LGALS3^+^ plaque cells and reduced plaque size, suggesting that SMC-derived SMA^−^ cells have deleterious effects in atherosclerosis^3^. Cap SMCs are widely implicated in stabilizing plaques against rupture, but our results suggest that these cells also have a negative impact on plaque dynamics by giving rise to pathological SMC marker^−^ core cells. Strategies to target SMC marker^+^ cap cells and prevent them from transdifferentiating and migrating into the core are needed.

In an atheroprone background (*ApoE*^(−/−)^ or *Ldlr*^(−/−)^), deletion of *Itgb3* exacerbates HFD-induced atherosclerosis^16, 17^, but underlying mechanisms are not well understood. Herein, we demonstrate that in SMC-derived cells, integrin β3 is expressed on SMC marker^+^ cap cells but not on core cells whereas CD36 is primarily expressed in core cells (Fig. 5 and Supplementary Fig. 8). Integrin β3 has been implicated in regulating SMC migration and proliferation^18–20, 38, 39^ but not marker expression. The aortic media and plaque of high fat-fed *ApoE*^(−/−)^, *Itgb3*^(−/−)^ mice harbor many cells expressing markers of both SMCs (SMMHC) and macrophages (CD68), and knockdown of Itgb3 in *ApoE*^(−/−)^ SMCs induces scavenger receptor Cd36 expression and ox-LDL uptake and facilitates cholesterol-induced transdifferentiation to a macrophage-like phenotype (Fig. 5). TLRs are the best characterized class of pattern-recognition receptors of the innate immune system, and recently, ox-LDL treatment of human arterial SMCs have been shown to induce TLR4 expression^40^. Herein, we demonstrate that knockdown of Itgb3 enhances Tlr4 levels in SMCs, and in the context of Itgb3 knockdown, Tlr4 reduction attenuates both Cd36 expression and cholesterol-induced transdifferentiation to a macrophage-like phenotype. Taken together, we speculate that in the early plaque, downregulation of integrin β3 in SMA^+^ cap cells or their progeny facilitates migration into the core, TLR4-induced CD36 expression, cholesterol uptake, and transdifferentiation.

No molecular or cellular players have previously been identified that regulate clonality of smooth muscle-derived cells in atherosclerosis. Our studies demonstrate that in contrast to *Itgb3*^(+/+)^, *ApoE*^(−/−)^ mice, multiple pre-existing SMCs are recruited into the plaques of *Itgb3*^(−/−)^, *ApoE*^(−/−)^ mice (Fig. 6). Transplant of *Itgb3*^(−/−)^, *Ldlr*^(−/−)^ bone marrow into *Itgb3*^(+/+)^, *Ldlr*^(−/−)^ mice worsens HFD-induced atherosclerosis^17^, and remarkably, we show that transplant of *Itgb3*^(−/−)^, *ApoE*^(−/−)^ bone marrow into *Itgb3*^(+/+)^, *ApoE*^(−/−)^ mice results in polyclonal smooth muscle-derived plaque cells. These results indicate that *Itgb3* null bone marrow-derived cells (most likely macrophages) are integral in the recruitment of multiple SMCs into the atherosclerotic plaque. There is a dearth of investigations into the potentially critical effects macrophages and macrophage-derived factors play in modulating the biology of SMCs in general and especially in the context of atherosclerosis. Our findings with isolated cells indicate that medium conditioned by *Itgb3*^(−/−)^ macrophages induces substantially more migration and proliferation of aortic SMCs compared to medium conditioned by macrophages wild type for *Itgb3*. Interestingly, integrin β3 is not detectable in macrophages of early plaques by immunohistochemistry (Supplementary Fig. 8). Thus, we speculate that *Itgb3*^(−/−)^ monocytes are altered such that when they enter the plaque and become macrophages, their gene expression differs from wild-type macrophages, and this difference includes secreted factors that directly induce multiple SMCs to migrate into the lesion and proliferate. An alternative or additional plausible explanation for the in vivo findings is that *Itgb3*^(−/−)^ bone marrow-derived cells signal to non-smooth muscle cells in the blood vessel/plaque milieu, such as endothelial cells, that in turn activate SMCs.

In sum, the increased number of recruited SMC progenitors and altered fate of these cells in *Itgb3* mutant mice are likely major factors exacerbating atherosclerosis. More broadly, we speculate that the number of smooth muscle-derived progenitors recruited into a plaque is a key determinant of the rate of progression of atherosclerosis. Thus, therapeutic approaches that reduce the recruitment and alter the fate of such progenitors into human atherosclerotic plaques warrant intense investigation.

## Methods

### Animals

Mouse experimental protocols were approved by the IACUC at Yale University, and we complied with all relevant ethical regulations. Wild type (C57BL/6, FVB), *ApoE*^(−/−)^, *ROSA26R*^(*CreER*/*CreER*)^, *ROSA26R*^(*mTmG*/*mTmG*)^, *Itgb3*^(−/−)^, and X-linked *D4*/*XEGFP* (denoted herein as *X*^*GFP*^) mice were purchased from Jackson Laboratories^23, 26, 41, 42^. Mice carrying an inducible Cre recombinase, *SMMHC*-*CreER*^*T2*^, *SMA*^-^*CreER*^*T2*^, *Csfr1*-*Mer*-*iCre*-*Mer*, or the multi-color Rainbow (Rb) Cre reporter *ROSA26R*^*Rb*^ have been described^22, 29, 35, 43, 44^. X-chromosome inactivation studies with *X*^*GFP*^*X*^+^ mice are limited to females, whereas experiments with mice carrying the *SMMHC*-*CreER*^*T2*^ transgene are restricted to males as this transgene incorporated on the Y-chromosome^44^. Otherwise, studies utilized both male and female mice. For timed pregnancies, noon of the day of vaginal plug detection was designated as E0.5, and embryos were dissected in phosphate-buffered saline (PBS). For atherosclerosis studies, ~2-month-old mice were fed a HFD (40% fat by calories, 1.25% cholesterol by weight) for up to 16 weeks.

### Human tissue

All procedures involving human subjects were approved by the Institutional Review Boards of Yale University (IRB #9908011041) and the New England Organ Bank, and we complied with all relevant ethical regulations. Human left main coronary artery samples were obtained from explanted hearts of transplant recipients or cadaver organ donors in the operating room and fixed in formalin^45^. The arteries had macroscopic atherosclerotic disease which was confirmed by microscopy. For surgical patients, a waiver for consent was approved, and for deceased organ donors, a family member provided informed consent.

### Immunohistochemistry

Following euthanasia, embryos, pups, or dissected adult aortas were fixed in 4% paraformaldehyde for 2 h at 4 °C. Tissue was then incubated in 30% sucrose, embedded in optical cutting temperature compound (Tissue Tek), and stored at −80 °C. Tissue was cryosectioned (10 µm) in the transverse axis, and sections were incubated with blocking solution (5% goat serum in 0.1% Triton X-100 in PBS (PBS-T)) and then with primary antibodies diluted in blocking solution overnight at 4 °C. On the next day, sections were washed with PBS-T and then incubated with secondary antibodies for 1 h. Primary antibodies used were anti-CD31 (1:500, BD Pharmingen, 553370), anti-SMMHC (1:100, Biomedical Technologies, J64817), anti-GFP (1:500, Invitrogen, A11122; 1:100, Abcam, ab13970), anti-pH3 (1:200, Millipore, 06–570), anti-Ki67 (1:100, Vector Labs, VP-RM04), anti-integrin β3 (1:200, Abcam, ab75872 and ab197662), anti-CD68 (1:200, Bio-Rad, MCA1957), anti-CD36 (1:100, Novus Biologicals, NB400-144), directly conjugated Cy3 anti-SMA (1:500, Sigma-Aldrich, C6198), anti-PDGFR-β (1:100, Abcam, ab32570), and biotinylated anti-PDGFR-β (1:50, R&D, AF1042). For amplification of the PDGFR-β signal, sections were incubated with the biotin-conjugated antibody and then with the ABC Elite reagent (vector), and FITC Tyramide reagent (Perkin Elmer, SAT701001EA)^35^. Secondary antibodies were conjugated to either FITC or Alexa-488, −564, −647 fluorophores (1:500, Invitrogen). Nuclei were visualized by DAPI staining (1:1000, Sigma-Aldrich, D9542). Note, membrane-localized tdTomato (mT) fluorescence in the aorta of *ROSA26R*^(*mTmG*/+)^ mice is very weak and essentially undetectable in comparison to the strong fluorescence in the red channel of staining regimens with antibodies directed against SMA or SMMHC.

Left main coronary artery atherosclerosis specimens from adult patients (55 ± 11 years old; 2/3 men, 1/3 women) were fixed in formalin, paraffin-embedded, and sectioned^45^. Sections of human arteries were treated with xylene to remove paraffin, washed with ethanol, and then rehydrated into water. Rehydrated sections were washed with TNT (10 mM Tris-HCl, pH 8.0, 150 mM NaCl, and 0.2% Tween-20) and incubated for 20 min in boiling 10 mM sodium citrate, pH 6.0 for antigen retrieval. Sections were then immersed in cold water and immunostained as described above for cryosections except washes were done in TNT. As previously described^45^, the ratio of the thickness of the intima (I) to media (M) as measured on H&E and Movat-stained slides was used to assess atherosclerosis severity. No or mild atherosclerosis was classified as I:M less than 0.2; moderate disease corresponded to I:M between 0.2 and 1.0; and an I:M greater than 1.0 was classified as severe atherosclerosis.

### Clonal analysis during normal aorta morphogenesis

Dams pregnant with *ROSA26R*^(*CreER*/*Rb*)^ embryos were induced with a single intraperitoneal injection of 4-OH-T at E5.25, and then embryos were analyzed at E14.5, E16.5, or as adults (5 weeks). Serial 10-µm-thick transverse sections through the distal thoracic descending aorta (600 µm) were stained with DAPI and imaged using fluorescent filters for DAPI and the Rb colors (i.e., Cerulean, mOrange, and mCherry). Each marked cell in the aortic wall and lung mesenchyme was scored based on color. High doses of 4-OH-T (150 µg) yielded mice invariably (100%) with multi-color marked cells in both the aortic wall and the lung mesenchyme (*n* = 5). Of the embryos (*n* = 28) induced at limiting 4-OH-T doses (20–100 µg), in terms of the aortic wall, 39% had no marked cells, 54% were labeled with a single color, indicating clonality and only rarely (*n* = 2) were marked cells multi-color (i.e., polyclonal; 7%). With regard to the lung mesenchyme, these limiting 4-OH-T doses yielded 57% of embryos lacking marked cells, 36% with labeled cells of a single color, and only 7% with multi-color cells. These studies included 17 embryos (see Supplementary Table 1) with both: (i) single labeled cells in one of the tissues (i.e., aortic wall or lung mesenchyme) and (ii) in the other tissue, either no marked cells or single labeled cells. Only four of these 17 embryos had labeled cells of the same color in the aortic wall and lung mesenchyme.

In addition, for longitudinal aortic sections, dams pregnant with *ROSA26R*^(*CreER*/*Rb*)^ embryos were induced with a single limiting 4-OH-T dose (20 µg) at E5.25, and then embryos were allowed to develop and were analyzed during adulthood (8 weeks). Serial 10-µm-thick longitudinal sections of the distal thoracic descending aorta (320 µm) were stained and imaged as described for transverse sections above. Of the embryos induced (*n* = 10), 60% had no marked cells and 40% were labeled with a single color (Supplementary Table 1). Per each marked cell or group of contiguous marked cells, we scored the number of contiguous marked cells.

Dams pregnant with *SMMHC*-*CreER*^*T2*^ embryos that were also heterozygous for a Cre reporter (i.e., *ROSA26R*^(*mTmG*/+)^ or *ROSA26R*^(*Rb*/+)^) were induced with a single limiting tamoxifen (0.5 mg) injection at E9.5. Embryos were allowed to develop until E14.5 or E16.5 when the distal descending aorta was sectioned (60 consecutive sections of 10 µm thickness), stained (with DAPI and in the case of GFP clones, also for GFP, SMA, and CD31), and analyzed. Each marked cell was scored based on position and specific layer in the aorta and, in the case of the Rb Cre reporter, on color (i.e., Cerulean, mOrange, or mCherry). Of all embryos analyzed (*n* = 29), 48% had aortas containing marked cells (*n* = 14, see Supplementary Table 2) while 52% of aortas were not labeled. Of the *SMMHC*-*CreER*^*T2*^, *ROSA26R*^(*Rb*/+)^ embryos analyzed with marked aortic SMCs (*n* = 7), all of them were of a single color. Higher tamoxifen doses (0.6–2 mg) resulted in more embryos with marked cells (74%), frequent multi-color labeling (67%) and more cells marked. In contrast, lower doses (0.125–0.4 mg) did not induce any marked aortic cells (*n* = 23).

### Fate and clonal analysis and proliferation in atherogenesis

Adult *ApoE*^(−/−)^ mice that were wild type or null for *Itgb3* and also carrying an inducible Cre recombinase (*SMMHC*-*CreER*^*T2*^, *SMA*-*CreER*^*T2*^, or *Csf1r*-*Mer*-*iCre*-*Mer*) and a Cre reporter (*ROSA26R*^(*mTmG*/+)^ or *ROSA26R*^(*Rb*/+)^) were induced with tamoxifen (1 mg/day) for five days for *SMMHC*-*CreER*^*T2*^ or *SMA*-*CreER*^*T2*^ mice or for 20 days for *Csf1r*-*Mer*-*iCre*-*Mer* mice. Mice were then rested for five days, fed a HFD for up to 16 weeks, and then euthanized. Serial sections through the aortic root were stained in the case of the *ROSA26R*^*mTmG*^ reporter for GFP, SMA and nuclei (DAPI), and in proliferative studies for the mitotic marker phosphohistoneH3 (pH3) as well. Sections from *ROSA26R*^(*Rb*/+)^ mice were stained with DAPI and directly imaged using fluorescent filters for Cerulean, mOrange, and mCherry. For cell labeling quantification, marked cells in the aortic root media and plaque were scored for expression of Cerulean, mOrange, or mCherry. In all quantitative cellular studies, total cells were determined by counting DAPI^+^ nuclei.

For proliferation studies, pH3^+^ aortic root plaque cells of high fat fed *ApoE*^(−/−)^, *SMMHC*-*CreER*^*T2*^, *ROSA26R*^(*mTmG*/+)^ mice were scored as to their location in the core or cap and to whether or not they express GFP. In addition, cap cells were scored as SMA^+^ or SMA^−^. For mice maintained on HFD for 12 or 16 weeks, the cap of the plaque was defined as the region within 30 µm of the lumen boundary as previously classified by Gary Owens and colleagues^3^ (consisting of ~1/6 of the total plaque width) and the remaining plaque was designated as the core. Because *ApoE*^(−/−)^ mice maintained on a HFD for only 6 weeks have smaller plaques (total width ~90 µm), the cap was considered within 15 µm of the lumen boundary (again, ~1/6 of the total width). Similarly, human coronary artery samples with moderate or severe atherosclerosis were stained for the proliferation marker Ki67, and Ki67^+^ cells were scored as to their location within the cap (i.e., 1/6 of the total plaque width) or the core.

### Aortic SMC isolation

Aortic SMCs were isolated by modifications of procedures described previously^46^. Briefly, aortas from the root to the iliac bifurcation were dissected from adult *ApoE*^(−/−)^ mice and opened longitudinally, and endothelial cells and the adventitia were gently removed. The aortas were sequentially washed with 1% penicillin/streptomycin in PBS and then with 100% fetal bovine serum (FBS). Washed aortas were cut into small pieces and cultured in plastic dishes in DMEM supplemented with 20% FBS, 1% penicillin/streptomycin. After 3 days, the medium was replaced by fresh medium that was similar except with 10%, instead of 20%, FBS. SMCs that migrated out of the aortic pieces and adhered to the dish were trypsinized, expanded, and passaged. For experiments, cells were used until the fifth passage.

### siRNA-mediated knockdown of Itgb3

Isolated murine aortic SMCs were transfected with Lipofectamine RNAiMAX (Invitrogen) containing siRNAs (Origene) targeted against Itgb3 (50 nM) or scrambled RNA for 6 h. Cells were then washed in PBS and cultured in DMEM supplemented with 10% FBS for 72 h.

### Loading with ox-LDL or water-soluble cholesterol

Following Itgb3 knockdown, SMCs were serum starved in DMEM with 0.2% BSA for 24–48 h and then loaded with LDL or cholesterol. For LDL experiments, SMCs were incubated with DiI-conjugated-LDL or ox-LDL (10 μg/ml, Alfa Aesar), 0.2% BSA in DMEM for 10 h at 37 °C, washed with PBS, and then fixed with 3% paraformaldehyde. For water-soluble cholesterol experiments, SMCs were cultured with cholesterol:methyl-β-cyclodextrin complexes (10 µg/ml, Sigma), 0.2% BSA in DMEM for 3 days, and then lysed for RNA isolation.

### Quantitative real-time PCR analysis

RNA was isolated from aortic SMCs of *ApoE*^(−/−)^ mice with the PureLink^TM^ RNA Minikit (Invitrogen), and this RNA (0.2 µg) was reverse transcribed with the iScript cDNA Synthesis Kit (Biorad). The expression levels of selected genes were determined by quantitative PCR and normalized to *Gapdh*. The following forward and reverse primer pairs were used for these genes (encoded protein): *Abca1* (ABCA1) - GGTTTGGAGATGGTTATACAATAGTTGT and CCCGGAAACGCAAGTCC; *Cd36* (CD36) - GGACATTGAGATTCTTTTCCTCTG and GCAAAGGCATTGGCTGGAAGAAC; *Csf1r* (CSF1R) - TGGATGCCTGTGAATGGCTCTG and GTGGGTGTCATTCCAAACCTGC; *Gapdh* (GAPDH) - CATCACTGCCACCCAGAAGACTG and ATGCCAGTGAGCTTCCCGTTCAG; *Itgam* (CD11b/MAC1) - TACTTCGGGCAGTCTCTGAGTG and ATGGTTGCCTCCAGTCTCAGCA; *Itgb3* (Integrin β3) - GTGAGTGCGATGACTTCTCCTG and CAGGTGTCAGTGCGTGTAGTAC; *Klf4* (KLF4) - CTATGCAGGCTGTGGCAAAACC and TTGCGGTAGTGCCTGGTCAGTT; *Lamp2* (LAMP2/MAC3) - GAGCAGGTGCTTTCTGTGTCTAG and GCCTGAAAGACCAGCACCAACT; *Lgals3* (LGALS3/MAC2) - AACACGAAGCAGGACAATAACTGG and GCAGTAGGTGAGCATCGTTGAC; *Ldlr* (LDLR) - GGTACTGGCAACCACCATTGGG and GCCAATCGACTCACGGGTTCAG; *Lrp1* (LRP1) - TTCAGACGAACGCGGGTGCC and CGGGCGACAGCGGCACTTAA; *Myh11* (SMMHC) - AAGCTGCGGCTAGAGGTCA and CCCTCCCTTTGATGGCTGAG; *Smtn* (Smoothelin) - CACGTCACTACCTTCAGCCATG and TGCGCCATTAGCTGCTTCCACT; *Tlr4* (TLR4) AGCTTCTCCAATTTTTCAGAACTTC and TGAGAGGTGGTGTAAGCCATGC.

### Peripheral blood cell counts

Blood was collected by retro-orbital puncture in heparinized micro-hematocrit capillary tubes. Total leukocyte with differential, red blood cell and platelet counts were performed using Hemavet hematology analyzer.

### Bone marrow transplant

*Itgb3*^(+/+)^, *ApoE*^(−/−)^, *SMMHC*-*CreER*^*T2*^, *ROSA26R*^(*Rb*/+)^ recipient mice were induced with tamoxifen (1 mg/day for five days), rested for five days, and then their bone marrow was lethally irradiated with a double dose of 550 rads (5.5 Gy) from a X-RAD 320 unit (Precision X-ray) 4 h apart before transplantation. Bone marrow was collected from femurs of experimental *Itgb3*^(−/−)^, *ApoE*^(−/−)^ or control *Itgb3*^(+/+)^, *ApoE*^(−/−)^ donor mice by flushing with sterile Opti-MEM medium (Thermo Fisher). Each recipient mouse was injected retro-orbitally with 2 × 10^6^ bone marrow cells. Four weeks after bone marrow transplantation, mice were switched to a HFD for up to 12 weeks and then euthanized. Transverse sections of the aortic root were stained, imaged, and quantified as noted above for clonal analysis.

### Macrophage-conditioned medium

Bone marrow from the femurs of experimental *Itgb3*^(−/−)^, *ApoE*^(−/−)^ or control *Itgb3*^(+/+)^, *ApoE*^(−/−)^ mice was harvested and cultured in Iscove’s Modified Dulbecco’s Medium supplemented with 20% FBS and 20% L-929 cell-conditioned medium^47, 48^. After 7 days in culture, contaminating non-adherent cells were removed. Adherent cells were cultured in RPMI, 10% FBS for 12 h, and then this macrophage conditioned medium was collected and used immediately or stored at −80 °C.

### Proliferation and migration assays of cultured SMCs

For proliferation studies, *ApoE*^(−/−)^ aortic SMCs were cultured in DMEM, 10% FBS, 1% penicillin/streptomycin until they reached ~60% confluency. Cells were then serum starved in DMEM overnight followed by incubation with macrophage-conditioned medium for 8 or 48 h. During the last 8 h of this incubation, EdU (10 μm) from the Click-iT EdU Alexa Fluor 555 Imaging Kit (Thermo Fisher Scientific, C10338) was added to the medium. Cells were then fixed in 4% PFA and permeabilized in 0.5% PBS-T. EdU staining was performed as per the manufacturer’s instructions.

For migration studies, cell culture inserts (Ibidi), composed of two chambers flanking a central insert that prevents cell growth, were used. *ApoE*^(−/−)^ aortic SMCs were added to each chamber and allowed to attach and grow to confluency. Cells were serum starved overnight and washed prior to removal of the insert and then cultured in macrophage-conditioned medium for 8 h. The cell coverage of the area that was blocked by the insert was measured immediately after insert removal and 8 h later.

### Imaging

Images were acquired with Nikon microscopes (Eclipse 80i upright fluorescent or Eclipse TS100 inverted) or Leica SP5 confocal microscope. For image processing, analysis and cell counting, Adobe Photoshop and Image J software were used.

### Statistical analysis

Student’s *t*-test, one-way ANOVA with Tukey’s multiple comparisons test, and Chi-square test were used to analyze the data (Prism7 software). Statistical significance threshold was set at *p* ≤ 0.05. Tests assumed normal distribution and were two-sided, and all data are presented as mean ± standard deviation.

### Data availability

All available data are available from the authors.

## Electronic supplementary material

Supplementary Information
