# Supplementary material for: Integrin beta3 regulates clonality and fate of smooth muscle-derived atherosclerotic plaque cells
Source: Nat Commun. 2018 May 25;9:2073. doi: 10.1038/s41467-018-04447-7 (PMC5970166; doi:10.1038/s41467-018-04447-7)
Supplement: Supplementary file 1 — Supplementary Information [file 41467_2018_4447_MOESM1_ESM.pdf]

SUPPLEMENTARY INFORMATION

Integrin beta3 regulates clonality and fate of smooth muscle-derived atherosclerotic plaque cells

Ashish Misra, Zhonghui Feng, Rachana R. Chandran, Inamul Kabir, Noemi Rotllan, Binod Aryal, Abdul Q. Sheikh, Ling Ding, Lingfeng Qin, Carlos Fernández-Hernando, George Tellides, Daniel M. Greif

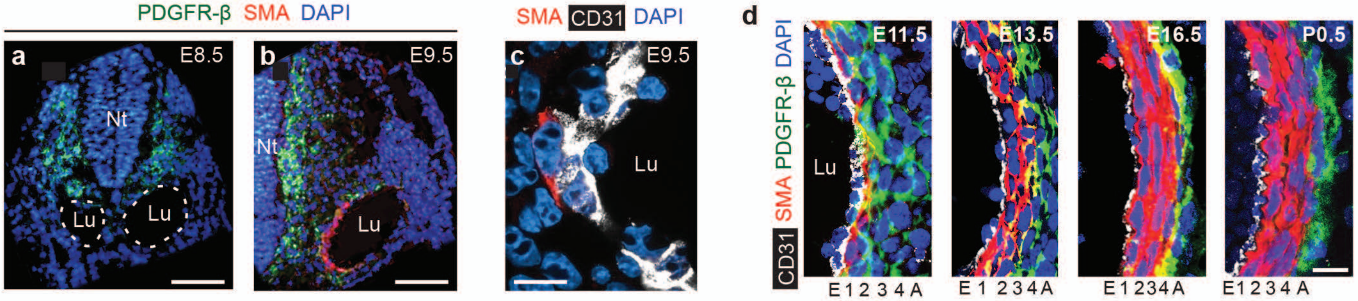

Supplementary Figure 1. Aortic SMCs are radially patterned. Transverse sections of wild type embryos at indicated ages and pup at P0.5 were stained for SMA, DAPI as well as PDGFR-β and/or CD31 (EC marker). **a,** At E8.5, the paired dorsal aortic EC tubes lack a SMC coating. **b, c,** By E9.5, SMA is initially expressed in PDGFR-β^+ cells adjacent to the ECs on the medial aspect of the dorsal aorta. **d,** Timeline of marker expression at E11.5 - P0.5 is shown. At E11.5, PDGFR-β^+SMA$^+$ cells comprise the first layer of the aortic wall throughout its circumference. By E13.5, in the first layer, the expression of PDGFR-β is downregulated and SMA is upregulated whereas the second layer now consists of PDGFR-β^+SMA$^+$ cells. This process continues radially outward during development until at P0.5, the aortic wall consists of 4-5 PDGFR-β^-SMC marker$^+$ cell layers and an outer adventitial layer of PDGFR-β^+SMA$^-$ cells. In addition, during the differentiation process, round shaped wall cells elongate circumferentially. Results are representative of n=5. Nt, neural tube; Lu, aortic lumen; E, endothelial layer; 1-4, smooth muscle layers; A, adventitial layer. Scale bars, 100 µm (**a, b**) and 10 µm (**c, d**).

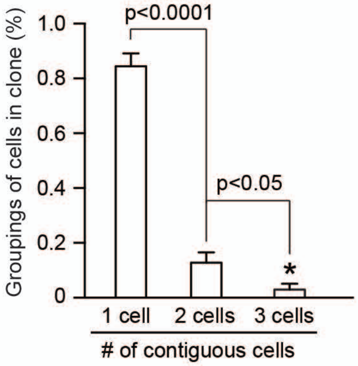

Average patch size: 1.2 ± 0.1 cells

Supplementary Figure 2. Patch size of aortic wall clones in longitudinal sections of *ROSA26R$^{(CreER/Rb)}$* adults. Dams pregnant with *ROSA26R$^{(CreER/Rb)}$* embryos were induced with a single threshold 4-OH-T dose (20 µg) injection at E5.25. Embryos were allowed to mature into adults (8 weeks) at which point longitudinal sections through the distal descending aorta were stained with DAPI and directly imaged for the Rb colors (see Methods and Fig. 1f). In each adult with marked cells (n=4 mice), all cells were a single color. Each marked cell or contiguous group of marked cells in a longitudinal section was scored as to the number of contiguous cells. The vast majority of patches were a single cell, and no patches were comprised of more than three contiguous cells. Per mouse, 21-41 cells were scored. One-way ANOVA with Tukey's multiple comparisons test, * vs. 1 cell, $p<0.0001$. Error bars represent standard deviations.

SMMHC-CreER clones

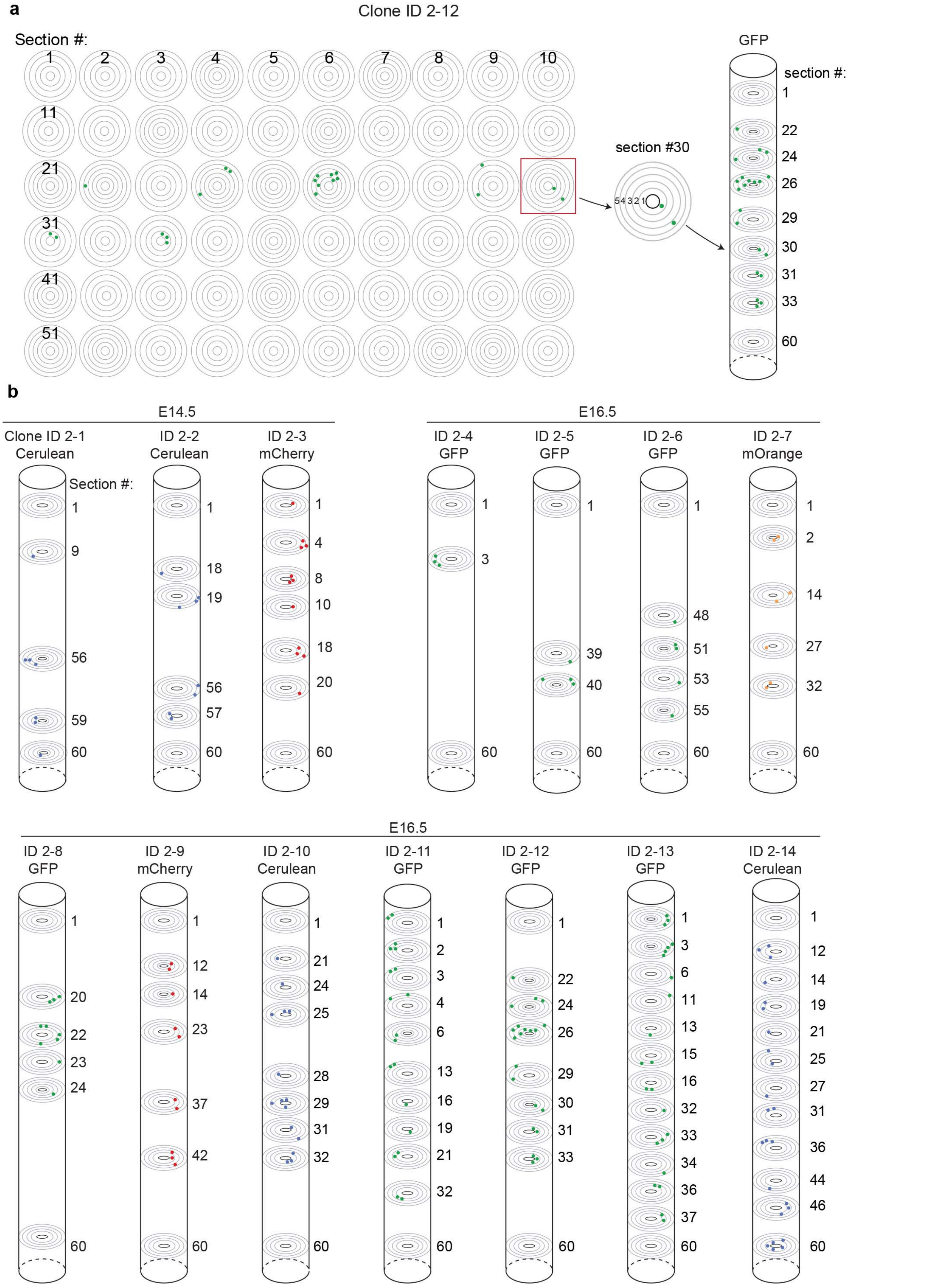

Supplementary Figure 3. Pattern of embryonic descending aortic wall clones derived from single initial inner layer SMCs. *SMMHC-CreER^{T2}* embryos also heterozygous for a Cre reporter (i.e., *ROSA26R$^{(mTmG/+)}$* or *ROSA26R$^{(Rb/+)}$*) were induced with a single injection of tamoxifen at the threshold dose (0.5 mg; see Methods and Supplementary Table 2) at E9.5 when aortic smooth muscle marker expression commences and is limited to inner layer SMCs. Sixty consecutive 10 µm cryosections in the distal descending aorta were stained for each embryo at E14.5 or E16.5. **a,** The position and color of marked cells are recorded for each section as shown in the schematic for clone ID2-12 in the left panel. A schematic of the 30th section is also shown with the numbers 1-5 indicating the smooth muscle layer (middle panel). In the right panel, the first and 60th section and all sections with marked cells are included in the columnar schematic for this clone. **b,** Similar columnar schematics for each of the *SMMHC-CreER^{T2}* clones (see Supplementary Table 2) are shown.

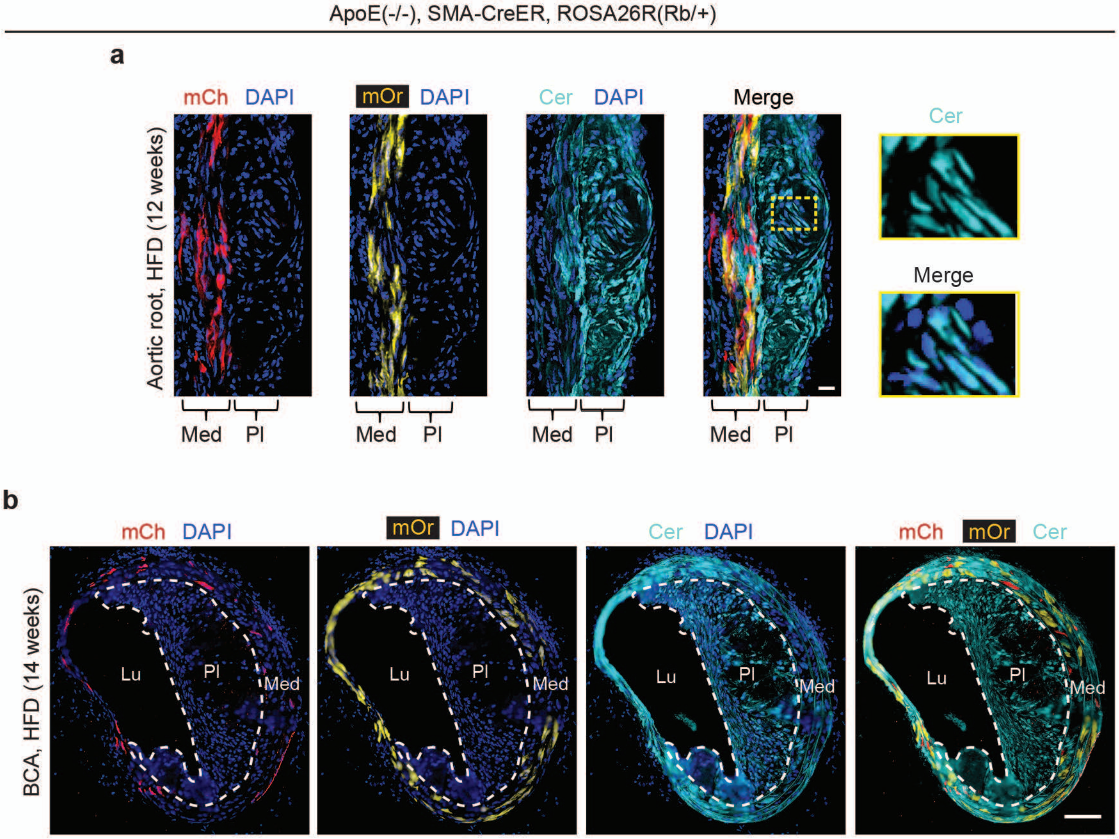

Supplementary Figure 4. The source of smooth muscle-derived cells in the atherosclerotic plaque is a single pre-existing SMC. *ApoE*$^{(-/-)}$, *SMA-CreER*^{T2}, *ROSA26R*$^{(Rb/+)}$ mice were induced with tamoxifen (1 mg/day for 5 days), rested (5 days), fed a high fat diet (HFD) and then transverse sections were stained with DAPI and directly imaged for the Rb colors (Cerulean [Cer], mCherry [mCh] or mOrange [mOr]). The media is comprised of a mixture of cells expressing Cer, mCh and mOr. All marked cells in the atherosclerotic plaque are a single color (in these two cases, Cer) indicating that a single SMC gives rise to all smooth muscle-derived plaque cells. **a**, Aortic root after exposure to HFD for 12 weeks is shown. Boxed region in the 4th panel is shown as a close-up on the right. Results are representative of n=9 mice, 11 plaques. **b,** Brachiocephalic artery after exposure to HFD for 14 weeks is shown. Area demarcated by the dashed line is the plaque; n=3 mice, 3 plaques. Med, tunica media; Pl, plaque; Lu, lumen. Scale bars, 10 μm (**a**) and 100 μm (**b**).

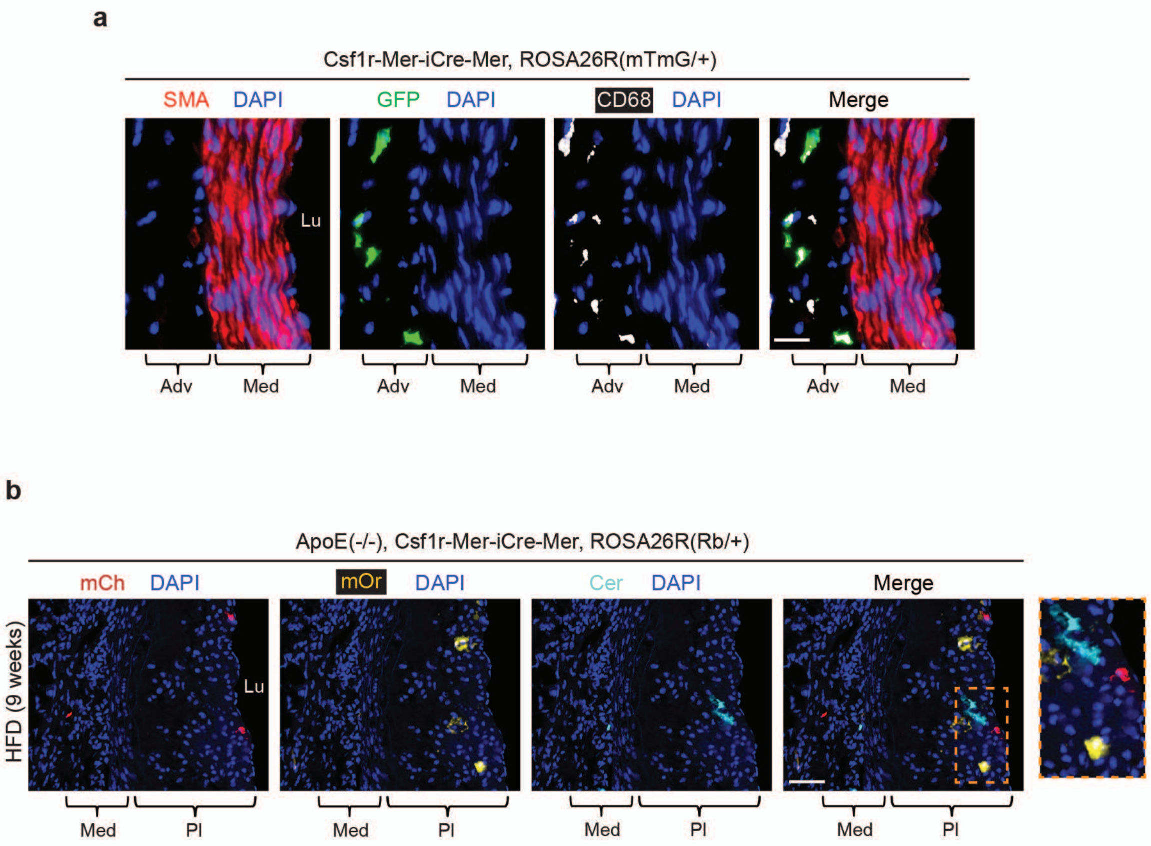

Supplementary Figure 5. Multiple cells marked by the inducible *Csf1r-Mer-iCre-Mer* are recruited into the atherosclerotic plaque. *ApoE$^{(-/-)}$, Csf1r-Mer-iCre-Mer* mice also carrying a Cre reporter were used to mark monocytes/macrophages. Transverse aortic root sections are shown. **a,** Mice also carrying *ROSA26R$^{(mTmG/+)}$* were induced with tamoxifen (1 mg/day for 20 days) and euthanized after a 5-day rest period. The section was stained for SMA, CD68, GFP and nuclei (DAPI). Note, the GFP^{+} cells do not co-localize with SMA^{+} cells but most express CD68. **b,** Mice also carrying *ROSA26R$^{(Rb/+)}$* were induced with tamoxifen, rested and then fed a high fat diet (HFD) for 9 weeks. Sections were stained with DAPI and directly imaged for the Rb colors [Cerulean (Cer), mOrange (mOr) and mCherry (mCh)]. The boxed region is shown on the right as a close-up. Marked cells in the atherosclerotic plaque are of different colors indicating that multiple marked cells are recruited into the plaque. Results are representative of n=3 mice (two female and one male). Lu, lumen; Adv, adventitia; Med, tunica media; Pl, plaque. Scale bars, 25 µm.

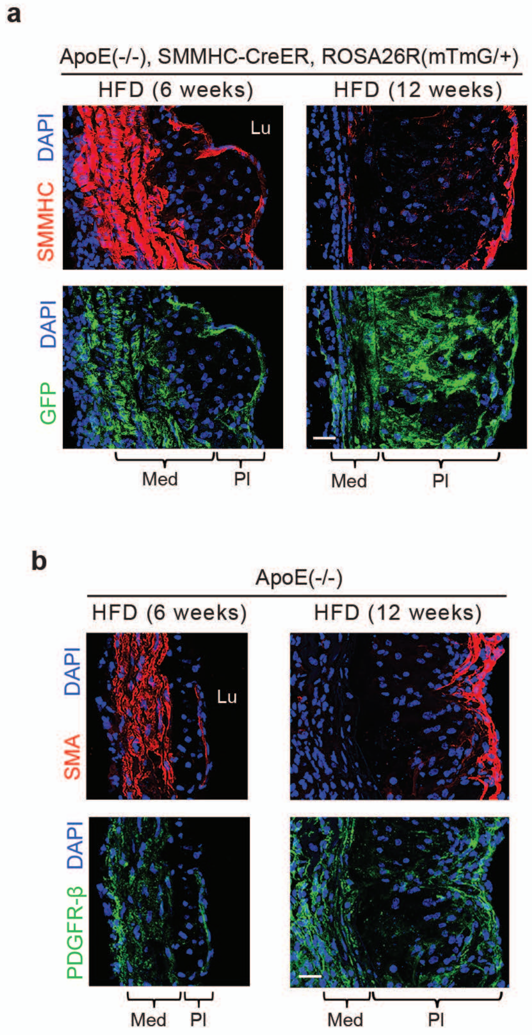

Supplementary Figure 6. Smooth muscle-derived cells express SMMHC in the cap but not in the core, and SMA$^+$ cap cells express PDGFR-β. Mice were fed a high fat diet (HFD) for 6 or 12 weeks and then euthanized, and transverse aortic root sections were stained. Merged images are shown in Figure 3c, d. **a,** *ApoE$^{(-/-)}$, SMMHC-CreER^{T2}, ROSA26R$^{(mTmG/+)}$* mice were induced with tamoxifen (1 mg/day) for five days, rested for five days and then maintained on a HFD. Sections were stained for GFP (fate marker), SMMHC and nuclei (DAPI). **b,** Aortic sections from high fat-fed *ApoE$^{(-/-)}$* mice were stained for PDGFR-β, SMA and nuclei (DAPI). Results are representative of n=5 mice Lu, lumen; Med, tunica media; Pl, plaque. Scale bars, 25 µm.

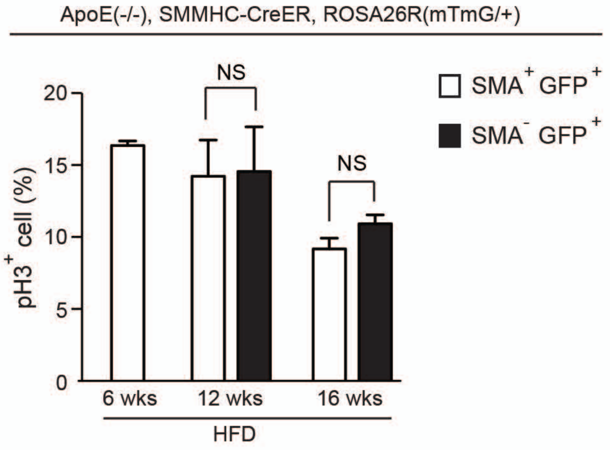

Supplementary Figure 7. Proliferation rates of smooth muscle-derived SMA^{+} and SMA^{-} cap cells are similar. *ApoE$^{(-/-)}$, SMMHC-CreER^{T2}, ROSA26R$^{(mTmG/+)}$* mice were induced with tamoxifen, rested and then fed a high fat diet (HFD) for 6, 12 or 16 weeks. Mice were euthanized and transverse aortic root sections were stained for GFP (fate marker), pH3 (mitotic marker), SMA and nuclei (DAPI). The percent of SMA^{+}GFP^{+} or SMA^{-}GFP^{+} cap cells that were pH3^{+} was quantified per HFD duration. Note that there is no bar for SMA^{-}GFP^{+} cells at 6 weeks of HFD because 99% of GFP^{+} cap cells are SMA^{+} at this time point. n=3 mice for each time point, and 10 sections per mouse were analyzed. Student's t-test; NS, not significant. Error bars represent standard deviations.

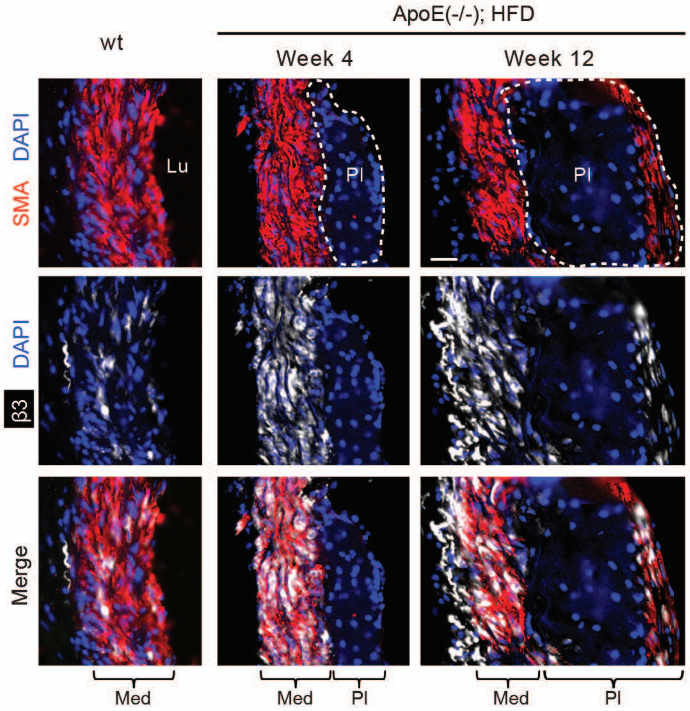

Supplementary Figure 8. Integrin β3 expression is upregulated in SMCs of the atherosclerotic aorta. Adult *ApoE*$^{(-/-)}$ mice were fed a high diet for 4 or 12 weeks, and wild type mice on a chow diet were included as a control. Transverse sections through the aortic root were stained for SMA, integrin β3 and nuclei (DAPI). β3 expression is increased in the atherosclerotic vessel, especially in the SMA^{+} cells of the tunica media and the cap. Results are representative of n=5 mice. Lu, lumen; Med, media; Pl, plaque. Scale bar, 25 μm.

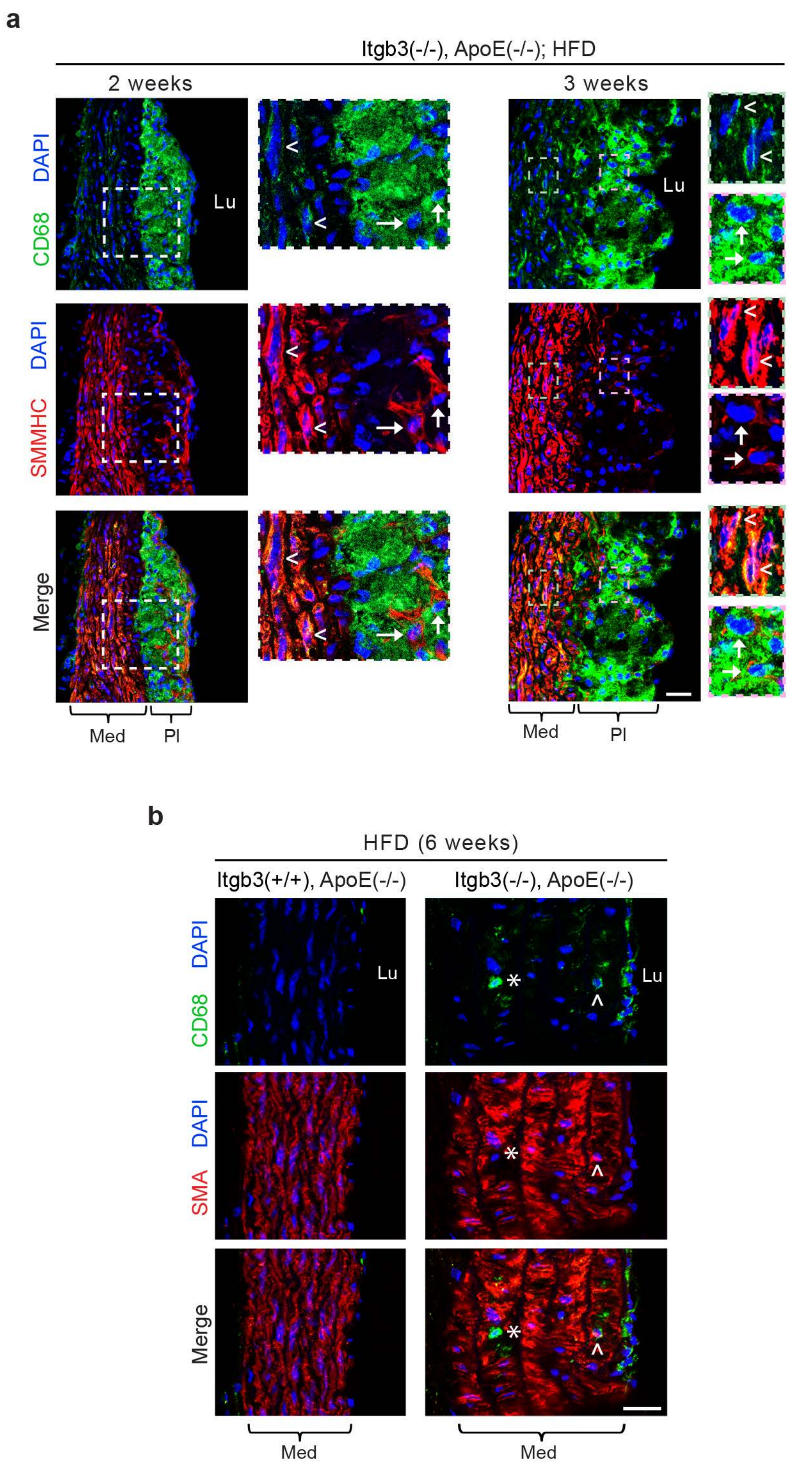

Supplementary Figure 9. In *Itgb3*$^{(-/-)}$*, ApoE*$^{(-/-)}$ mice fed a high fat diet, SMC marker^{+}CD68^{+} cells are common in the atherosclerotic plaque and adjacent media of the aorta but rare in the media that is distant from the plaque. *Itgb3*$^{(-/-)}$*, ApoE*$^{(-/-)}$ or *Itgb3*$^{(+/+)}$*, ApoE*$^{(-/-)}$ mice were fed a high fat diet (HFD) for 2, 3 or 6 weeks as indicated, and then transverse aortic sections were stained for CD68, nuclei (DAPI) and either SMMHC (**a**) or SMA (**b**). In **a**, plaques with adjacent media are displayed. Boxed regions are shown as close-ups on the right, and cells expressing SMMHC and CD68 in the media (arrowheads) and plaque (arrows) are indicated. In **b,** the media without a substantial adjacent plaque are shown, and arrowhead and asterisk indicate rare SMA^{+}CD68^{+} and SMA^{-}CD68^{+} cells in the media, respectively. Results are representative of n=3 mice. Lu, lumen; Med, tunica media; Pl, plaque. Scale bars, 25 µm.

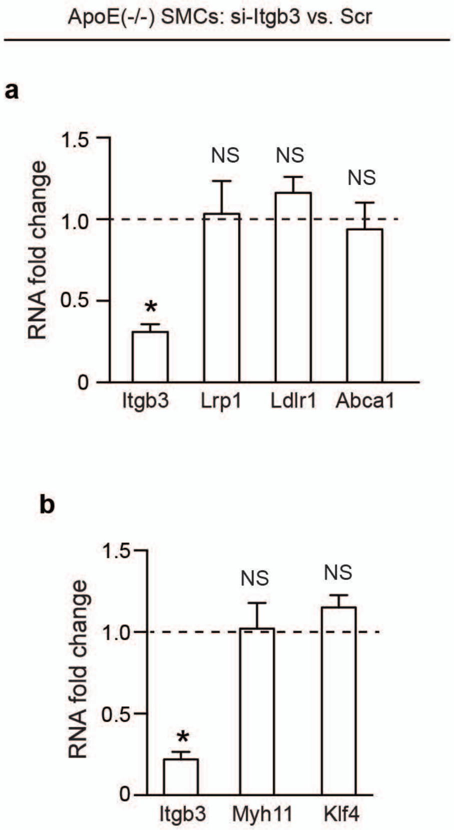

Supplementary Figure 10. Itgb3 knockdown in *ApoE*$^{(-/-)}$ SMCs does not alter expression levels of cholesterol receptors/transporters Lrp1, Ldlr and Abca1 or levels of Myh11 or Klf4. Aortic SMCs were isolated from *ApoE*$^{(-/-)}$ mice and treated with scrambled (Scr) or Itgb3 siRNA. SMCs were subjected to qRT-PCR for Itgb3 and either for Lrp1, Ldlr and Abca1 in **a** or for Myh11 and Klf4 in **b**. RNA levels are relative to Gapdh and normalized to Scr treatment; n=2-3 in duplicate. Student's t-test, * vs. Scr, $p<0.005$ and NS, not significant. Error bars represent standard deviations.

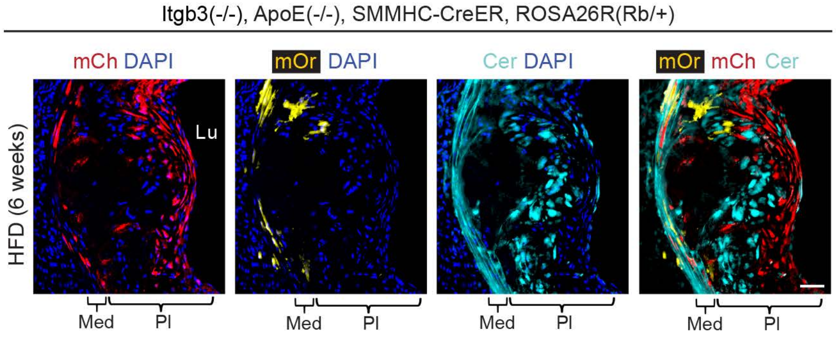

Supplementary Figure 11. Global knockout of *Itgb3* results in polyclonal SMC-derived atherosclerotic cells. *Itgb3*$^{(-/-)}$, *ApoE*$^{(-/-)}$, *SMMHC-CreER*^{T2}, *ROSA26R*$^{(Rb/+)}$ mice were induced with tamoxifen, rested and then fed a high fat diet (HFD) for 6 weeks. Transverse aortic root sections were stained for DAPI and directly imaged for the Rb colors (mCherry [mCh], mOrange [mOr] and Cerulean [Cer]). As in Figure 6a, this clone shows multiple patches of SMC-derived plaque cells of different colors with minimal mixing indicating that multiple SMMHC^{+} progenitors are recruited into the plaque and locally expand with limited intermingling between daughter cells of each progenitor. Results are representative of n=3 mice, 6 plaques. Lu, lumen; Med, tunica media; Pl, plaque. Scale bar, 25 μm.

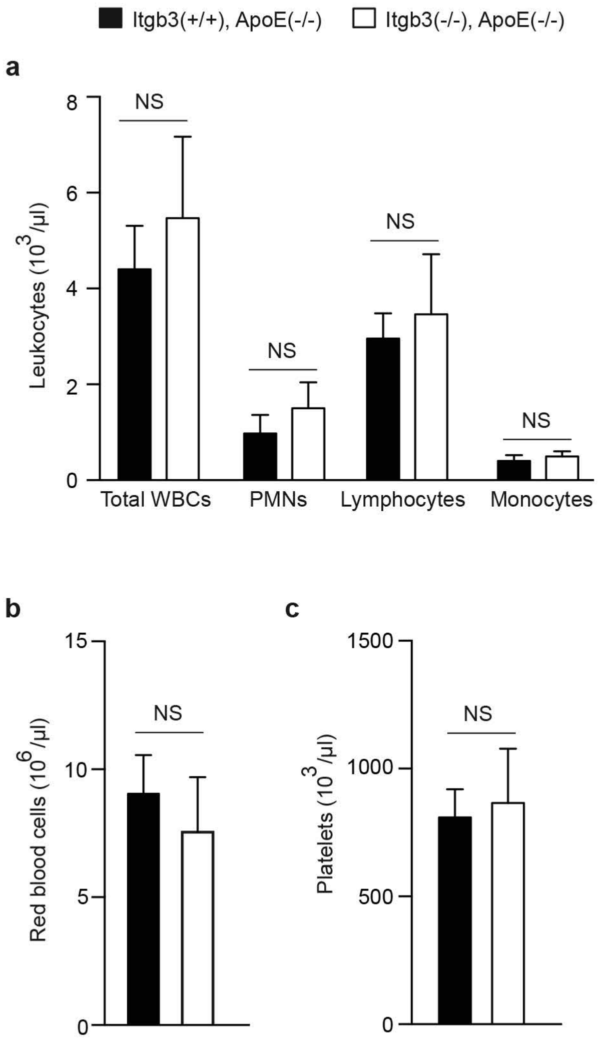

Supplementary Figure 12. Peripheral blood cell counts are similar for experimental *Itgb3$^{(-/-)}$, ApoE$^{(-/-)}$* and control *Itgb3$^{(+/+)}$, ApoE$^{(-/-)}$* donor mice. Peripheral blood from experimental and control donor mice was analyzed for quantity of white blood cells with differential (**a**), red blood cells (**b**) and platelets (**c**); n=6 experimental mice and n=5 control mice. Student's t-test with NS, not significant. Error bars represent standard deviations.

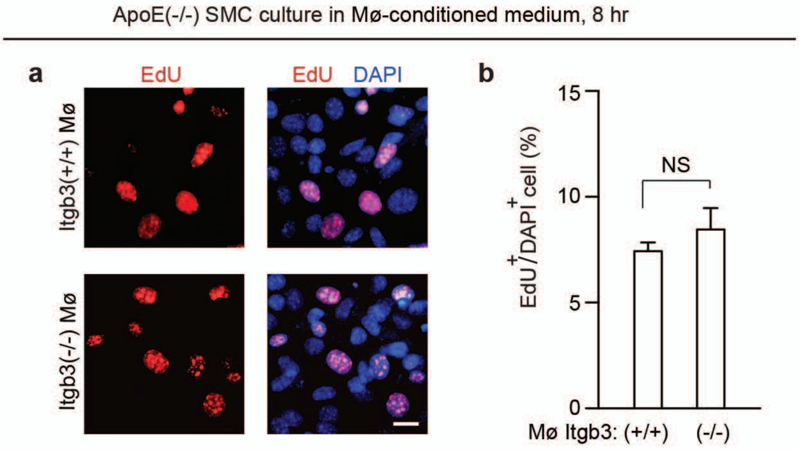

Supplementary Figure 13. The effects on *ApoE$^{(-/-)}$* SMC proliferation are similar for culturing for 8 hours with conditioned medium from bone marrow-derived macrophages of *Itgb3$^{(+/+)}$*, *ApoE$^{(-/-)}$* or *Itgb3$^{(-/-)}$*, *ApoE$^{(-/-)}$* mice. *ApoE$^{(-/-)}$* SMCs were cultured for 8 hours in the presence of EdU in medium conditioned by bone marrow-derived macrophages of *Itgb3$^{(+/+)}$*, *ApoE$^{(-/-)}$* or *Itgb3$^{(-/-)}$*, *ApoE$^{(-/-)}$* mice. **a,** SMCs were stained for EdU and nuclei (DAPI). **b,** The percent of DAPI^{+} nuclei that also express EdU was quantified from sections as in **a** n=3; Student's t-test with NS, not significant. Error bars represent standard deviations.

Supplementary Table 1

Clonal analysis of progenitors of descending aorta or lung mesenchyme by threshold 4-OH tamoxifen-induction of *ROSA26R(Rb/CreER)* mice at E5.5

Clone ID	4-OH Tamoxifen dose (μg)	Analysis age	Color of marked cells in: descending aorta	lung mesenchyme
**Transverse sections:**				
1-1	100	E14.5	mOrange	mCherry
1-2	100	E14.5	mCherry	mCherry
1-3	100	E14.5	Cerulean	Cerulean
1-4	100	E14.5	mCherry	None
1-5	100	E14.5	mCherry	None
1-6	100	E14.5	None	Cerulean
1-7	50	E14.5	mCherry	Cerulean
1-8	50	E14.5	Cerulean	Cerulean
1-9	50	E14.5	mCherry	Cerulean
1-10	50	E14.5	mCherry	None
1-11	50	E14.5	None	mOrange
1-12	50	5 weeks	mOrange	Cerulean
1-13	50	5 weeks	Cerulean	Cerulean
1-14	20	E16.5	mOrange	None
1-15	20	E16.5	mCherry	None
1-16	20	E16.5	Cerulean	None
1-17	20	E16.5	mCherry	None
**Longitudinal sections:**				
1-18	20	8 weeks	mCherry	Not assessed
1-19	20	8 weeks	mOrange	Not assessed
1-20	20	8 weeks	mOrange	Not assessed
1-21	20	8 weeks	mCherry	Not assessed

Supplementary Table 2

Clonal analysis of aortic SMCs by induction of *SMMHC-CreER* mice also carrying a Cre reporter with single injection of threshold tamoxifen dose (0.5 mg) at E9.5*

Clone ID	Analysis age	Cre reporter	Clone size	Clone marker	Doubling time
2-1	E14.5	Rainbow	7 cells	Cerulean	1.6 days
2-2	14.5	Rainbow	8	Cerulean	1.5
2-3	14.5	Rainbow	12	mCherry	1.3
2-4	16.5	mTmG	3	GFP	4.1
2-5	16.5	mTmG	4	GFP	3.3
2-6	16.5	mTmG	5	GFP	2.8
2-7	16.5	Rainbow	7	mOrange	2.3
2-8	16.5	mTmG	10	GFP	2.0
2-9	16.5	Rainbow	10	mCherry	2.0
2-10	16.5	Rainbow	15	Cerulean	1.7
2-11	16.5	mTmG	19	GFP	1.5
2-12	16.5	mTmG	21	GFP	1.5
2-13	16.5	mTmG	23	GFP	1.4
2-14	16.5	Rainbow	25	Cerulean	1.4
				Mean (± s.d)	2.0 ± 0.8 days

*For doubling time calculation, the time of intial recombination was taken as E10 (i.e., 12 hrs after tamoxifen injection)

Supplementary Table 3

Clonal analysis of SMC-derived atherosclerotic plaque cells in *ApoE(-/-), ROSA26R(Rb/+)* mice also carrying *SMMHC-CreER* or *SMA-CreER*

Clone ID	Inducible SMC-Cre	Duration of HFD (weeks)	Clone marker	Total plaque cells counted	Marked plaque cells counted	% marked of plaque cells
3-1	SMMHC-CreER	6	Cerulean	810	80	0.099
3-2	SMMHC-CreER	6	mOrange	960	44	0.046
3-3	SMMHC-CreER	6	Cerulean	1243	82	0.066
3-4	SMMHC-CreER	6	Cerulean	1021	113	0.111
					Mean (±s.d.) =	0.08 ± 0.03
3-5	SMA-CreER	12	mOrange	1257	541	0.430
3-6	SMA-CreER	12	Cerulean	1253	399	0.318
3-7	SMMHC-CreER	12	mOrange	1097	434	0.396
					Mean (±s.d.) =	0.38 ± 0.06
3-8	SMA-CreER	16	mCherry	1700	1114	0.655
3-9	SMA-CreER	16	mCherry	1984	1151	0.580
3-10	SMMHC-CreER	16	mOrange	1638	869	0.531
3-11*	SMMHC-CreER	16	Cerulean	1227	608	0.496
					Mean (±s.d.) =	0.57 ± 0.07

*In clone ID3-11, there was an additional 5 marked cells, all of which were mCherry+.

Supplementary Table 4

Clonal analysis of SMC-derived atherosclerotic plaque cells in
***Itgb3(-/-), SMMHC-CreER, ROSA26R(Rb/+)* mice after HFD for 6 weeks**

Clone ID	Total plaque cells counted	Marked plaque cells counted: mOrange	mCherry	Cerulean	Total	% marked of plaque cells
4-1	1878	550	75	273	898	0.478
4-2	1717	265	426	153	844	0.492
4-3	880	174	228	102	504	0.573
4-4	1420	87	631	281	999	0.704
4-5	1921	430	183	191	804	0.419
4-6	1500	459	338	207	1004	0.669
					Mean (±s.d.) =	0.56 ± .11
